# Taxonomic significance of morphological and molecular variation in Egyptian Malvaceae species

**DOI:** 10.1186/s12870-025-06609-4

**Published:** 2025-05-16

**Authors:** Osama G. Ragab, Doaa M. El Kholy, Sarah M. M. Elkhiat, Amaal H. Mohamed, Azza A. F. Khafagi

**Affiliations:** 1 Department of Botany and Microbiology, Faculty of Science (Boys Branch), Al-Azhar University, Cairo, 11884 Egypt; 2 Botany and Microbiology Department, Faculty of Science (Girls Branch), Al-Azhar University, Cairo, Egypt

**Keywords:** Malvaceae, Morphology, Pollen grain, SCoT, Numerical analysis

## Abstract

**Background:**

This study examined the morphological characters and molecular analysis of 15 species belonging to eight genera of the family Malvaceae, collected from different localities in Egypt. The study focused on investigating the relations between the species based on evidence of macro- and micro-morphological characters and molecular analysis. Since species identification and classification are highly dependent on morphological traits.

**Results:**

The macro- and micro-morphology characters were used in analyzing the relationship among the studied species viz., habit, and nature of stem, type of stipule, type of lamina, inflorescence type, staminal tube, stigma type, and pollen grain sculpture. Furthermore, with the SCoT technique, differences in molecular markers were detected. In the start codon targeted SCOT analysis, eight SCoT primers were independently used. The total number of amplified fragments by all primers in the studied species was 100 fragments, including 51 polymorphic fragments with a ratio of 51%. The highest polymorphism among the studied species 72.73% was observed with the SCoT-06 primer. The lowest polymorphism 16.67% was obtained in the SCoT-07 primer. In three SCoT primers, SCoT-01, SCoT-06, and SCoT-10, no unique fragments were detected, while the other five primers yielded one unique band. In addition, the results of the phylogenetic tree constructed based on the similarity coefficient revealed by SCoT analysis confirm the macro and micro-morphological analysis findings.

**Conclusion:**

Generally, these data of morphological characters and molecular analysis support the resolution and identification of closely related Malvaceae species and offer insight into their phylogenetic relationships. This, in turn, supports previous taxonomic revisions in Malvaceae.

**Supplementary Information:**

The online version contains supplementary material available at 10.1186/s12870-025-06609-4.

## Introduction

The Malvaceae Mallow family is represented by 244 genera and 4225 species [1]. It is a widely distributed family that mainly occurs in warm temperatures and tropical regions, not including the cold areas [2, 3]. In Egypt, Malvaceae’s species and genera have varied over time; [4] recognized 31 species within eight genera, while El-Hadidi & Araffa [5] Malvaceae represented 26 species belonging to 11 genera. At last, the number of species was updated to 32 species belonging to 10 genera [6].

The members of this family have much economic importance; they can be used as food such as *Abelmoschus esculentus* or okra, or as a condiment *Hibiscus sabdariffa*, ornamental such as *Abutilon*, *Alcea*,* Althaea*,* Hibiscus* and *Malva*, industry such as *Gossypium* and also family Malvaceae has medicinal uses owing to its mucilage, fixed and essential oils, some of these species that are commonly used in folk medicine include leaves and flowers of *Althaea officinalis*, and *Malva sylvestris* are used as protectors in respiratory and digestive system irritations and inflammations [7–9].

Taia [10] contributed a general view of Malvaceae and a taxonomic revision of the genus *Abutilon* in Saudi Arabia based on morphological description. Naskar and Mandal [11] studied a characterization of some common species of the family Malvaceae based on the morphology of selective characters such as hypocalyx, staminal tube, stigmata head, and trichome [12]. Erarslan and Koçyiğit [13] revealed the systematic importance of the morphological characteristics of the Malvaceae family. El-Taher et al. [14]. and Mostafa et al. [15]. created a taxonomic revision of the genus *Hibiscus* in Egypt based on thirty-six morphological characteristics, including vegetative, flowers, and fruit parts. Singh et al. [16]. studied the variations in the morphological attributes of four species of the genus *Sida* belonging to the family Malvaceae. Xiao et al. [17]. and Ibrahim et al. [18] contributed a mixture of quantitative and qualitative morphological and palynological traits to the identification and classification of 24 cultivars of *Hibiscus syriacus*.

On the other hand, palynological studies have frequently been used in the taxonomy and phylogeny of several taxa [19–21]. Several workers emphasized the importance of pollen grain morphology in plant systematics, especially [21–26]. The pollen morphology of Malvaceae was included in many studies [27–33] that examined and determined the morphology of the pollen grain of 11 cultivars belonging to two species of the genus *Hibiscus* in Egypt. Moreover, the pollen grain morphology of 20 species belonging to seven genera of Malvaceae from Saudi Arabia was described by Abdel Khalik et al. [34].

Patil et al. [35]. studied 52 diagnostic characters of root, stem, leaf, flower, fruit, and seed of 16 *Abelmoschus* taxa by cluster analysis. Shamso & Khattab [36] studied 103 macro and micro-morphological characters, including vegetative parts, pollen grains, and seeds of 64 taxa belonging to 32 genera of Malvaceae based on numerical analysis. Analysis of 71 macro and micro-morphological characters of four species belonging to the subfamily Malvoideae Malvaceae were collected from different regions in Egypt using the UPGMA clustering method [37]. The relationship between six *Abutilon* species collected from various localities in Saudi Arabia was obtained by comparing the results using numerical taxonomic methods using morphological characters [38–40]. Ibrahim et al. [41]. examined macro and micro-morphological characteristics of seeds from 49 species across 34 genera and seven subfamilies of the Malvaceae *s.l.*, and 73 recorded traits based on storage proteins by using the upgraded Win Clad and TNT cladistic programs, a cladogram was constructed which indicated a clear suggestion about the systematic position between the studied species.

Determining plant traits and conducting genetic characterization are essential steps in the development of effective plant breeding programs [42–46]. Different forms of molecular markers based on DNA have been used to evaluate genetic diversity in plant species. Start Codon Targeted SCoT is a novel technique for generating plant DNA markers developed based on the short-conserved area flanking the ATG start codon in plant genes [47]. Start Codon Targeted DNA SCoT marker is a highly operative, rapid, and simple tool for genetic description, and they are employed to classify and define the genetic range of countless plants [48, 49]. The SCoT marker has been successfully used to evaluate genetic variety and structure, to classify cultivars, and for quantitative mapping and DNA fingerprinting in different plant species, as represented in [47, 50–55].

Huixing et al. [56]. performed a combination of morphological and molecular data using SCoT markers for 97 randomly collected *Malva* species in Iran. In addition, ten SCoT primers and five ISSR primers were used to examine the genetic variations among the 21 species of subfamily Malvoideae [57]. Despite numerous morphological and palynological studies in Malvaceae, few have combined these data with molecular markers to assess intra-familial relationships, particularly within the Egyptian flora therefore, this study aimed to investigate the morphological and molecular characteristics of some species of Malvaceae growing in Egypt and use these characteristics to classify the family and solve some taxonomic problems.

## Materials and methods

### Materials

In the current study, 15 species of Malvaceae belonging to 9 genera, which were collected fresh from their natural habitats at different sites in Egypt, in addition to Herbarium specimens, were acquired from the Herbarium of Alexandria University, Faculty of Science and Herbarium of Desert Research Center CAIH, Cairo, Egypt. Locality and date information of the specimens investigated were given in Table 1, and identified by Prof. Dr. Dalia Goda Gabr, Professor of Plant Taxonomy, at Botany and Microbiology Department, Faculty of Science, Al-Azhar University, Cairo, Egypt. The scientific names of the plants were verified according to the Plants of the World Online website: https://powo.science.kew.org/. The collected materials were identified by comparison with specimens kept in the herbarium of the Agricultural Museum CAIM and Cairo University Herbarium CAI, along with the plant keys of [6, 58, 59]. The collected species were prepared as herbarium specimens and deposited at the Herbarium of Botany and Microbiology Department, Faculty of Science, Al-Azhar University Girls Branch.

**Table 1:** List of studied species, localities, and data of collection

No.	Species	Locality and date	Deposition no.
1	*Abutilon grandifolium* Willd. Sweet	El-Zoharia botanical Garden, El-Zamalek. 15/3/2021	81
2	*Abutilon pannosum* G. Forst. Schltdl.	Gebel Alba, Wadi Eikwan upstream, southeast of Halayeb Alexandria University Herbarium, Faculty of Science. 3/3/1998	82
3	*Abutilon theophrasti* Medik.	Kafr El-Dawar, El-Beheira. 7/7/2020	83
4	*Alcea rosea* L.	Nasr City, Cairo. 26/1/2021	84
5	*Malva multiflora* Cav. Soldano, Banfi & Galasso	Alexandria, Matrouh road. 13/2/2021	85
6	*Malva neglecta* Wallr.	Sinai, Sant Catherine. 28/6/2021	86
7	*Malva nicaeensis* All.	Kafr El-dawar, El-Beheira. 3/3/2020	87
8	*Malva parviflora* L.	North Coast-Matrouh Road. 13/2/2021	88
9	*Malva sylvestris* L.	North Coast-Matrouh Road. 13/2/2021	89
10	*Malvastrum coromandelianum* L. Garcke	El-Saff, Giza, Cairo. 15/8/2021	90
11	*Sida alba* L.	Kafr El-Dawar, El-Beheira. 7/7/2020	91
12	*Gossypium barbadense* L.	Kafr El-Dawar, El-Beheira. 7/10/2021	92
13	*Abelmoschus esculentus* L. Moench.	El-Sheikh Dergham, Damietta Road. 20/10/2020	93
14	*Hibiscus sabdariffa* L.	El-Sheikh Dergham, Damietta Road. 20/10/2020	94
15	*Hibiscus trionum* L.	Kafr El-Dawar, El-Beheira. 7/7/2020	95

## Methods

The morphological features of the studied species were described from fresh and herbarium specimens. For the morphological investigation, the means of three measurements per specimen were recorded. The vegetative and floral characters were recorded in a cumulative plate, which was set up in a specific manner to clarify the significance of the characters examined. Details of the floral parts were recorded, examined, and photographed using a binocular stereo microscope Olympus SZ40-PT in conjunction with incident light coupled with a digital camera to determine the variation among species. The terminology used for describing stigma types and leaf texture corresponds to Radford et al. [60]. and Bendre & Kumar [61].

For pollen grains examination by light microscope LM a rapid method of preparation was performed using 5% NaoH for about 10 min heating in a water bath, then 1% safranin solution was applied to stain the grains, after that the pollen grains were mounted in glycerin jelly into glass slides and observed under light microscope E40-0.65 using the 15x eye lens. Five measurements per specimen were taken to measure the polar axis PA, equatorial diameter ED, and PA/ED ratios. Pollen grains were transferred into specimen stubs, coated with a thin gold film using a JEOL JFC 1100E ion-sputtering device and photographed with an electron microscope JEOL ISM-IT200, operated at an accelerating voltage of 20 kV. The work was carried out in the Electron Microscope Unit, Faculty of Science, Alexandria University. The terminology used for describing the morphology of pollen grains is by Erdtman [62], El-Ghamery et al. [63], Abdel Khalik et al.. [34], and Abdo El Samad et al. [64].

## Molecular analysis

### DNA isolation

In this investigation, DNA analysis was performed using the Start Codon Target (SCoT) reaction. Total DNA was extracted from 15 samples of malvaceae by DNeasy Plant Kit (QIAGEN, Germany). Two grams of leaf samples from each species were extracted for DNA analysis, and NanoDrop estimated the extracted DNA concentration and quality.

- SCoT-PCR Reactions: In the Start Codon Target SCoT-PCR analysis, eight SCoT primers were used to recognize polymorphism Table 2. The amplification reaction was carried out in 20 µl reaction volume containing 10 µl Master Mix (sigma), 2 µl primer (10 pcmol), 2 µl template DNA (10 ng), and 6 µl d H_2_O, according to Collard & Mackill [47].
- Thermocycling Profile PCR: PCR amplification was performed in a Perkin-Elmer/Gene Amp^®^ PCR System 9700 (PE Applied Biosystems) programmed to fulfill 40 cycles after an initial denaturation cycle for 5 min at 94ºC. Each cycle consisted of a denaturation step at 94ºC for 45s, an annealing step at 50ºC for 50s, and an elongation step at 72ºC for 1 min. The primer extension segment was extended to 7 min at 72ºC in the final cycle.
- Detection of the PCR Products: The amplification products were determined by electrophoresis in a 1.5% agarose gel containing ethidium bromide 0.5ug/ml in 1X TBE buffer at 95 volts. PCR products were visualized on UV light and photographed using a Gel Documentation System (BIO-RAD 2000).

**Table 2:** List of primers and their nucleotide sequences

Primer Name	Sequence
SCoT-01	5’-ACGAC*ATG*GCGACCACGC-3’
SCoT-02	5’-ACC*ATG*GCTACCACCGGC-3’
SCoT-04	5’-ACC*ATG*GCTACCACCGCA-3’
SCoT-05	5’-CA*ATG*GCTACCACTAGCG-3’
SCoT-06	5’-CA*ATG*GCTACCACTACAG-3’
SCoT-07	5’-ACA*ATG*GCTACCACTGAC-3’
SCoT-9	5’-ACAATGGCTACCACCAGC-3’
SCoT-10	5’-ACAATGGCTACCACTACC-3’

### Numerical analysis

A total of 165 characters which resulted from morphological data and molecular analysis were scored and coded for creating data matrix used for numerical analysis. These comparative characters for studied species were analyzed and the fifteen species were grouped by means of Hierarchical Cluster analysis used Bray-Curtis similarity and Euclidean distance. Fifteen of species under study were used as Operational Taxonomic Units (OUT’s). A cluster analysis was conducted in order to demonstrate the relationships between fifteen studied species, by using statistical program PRIMER (Software, version 6.0) is selected for purposes of analyzing taxonomic data in order to construct systems of classification of plants and for calculation the degree of similarity and distance among species; this version provides (Bray-Curtis similarity and Euclidean distance). The grouping of Operational Taxonomic Units (OUT′s) produced from the analysis were examined and compared with the current taxonomic classification of the family.

## Results

### Vegetative morphology

**Fig. 1:**
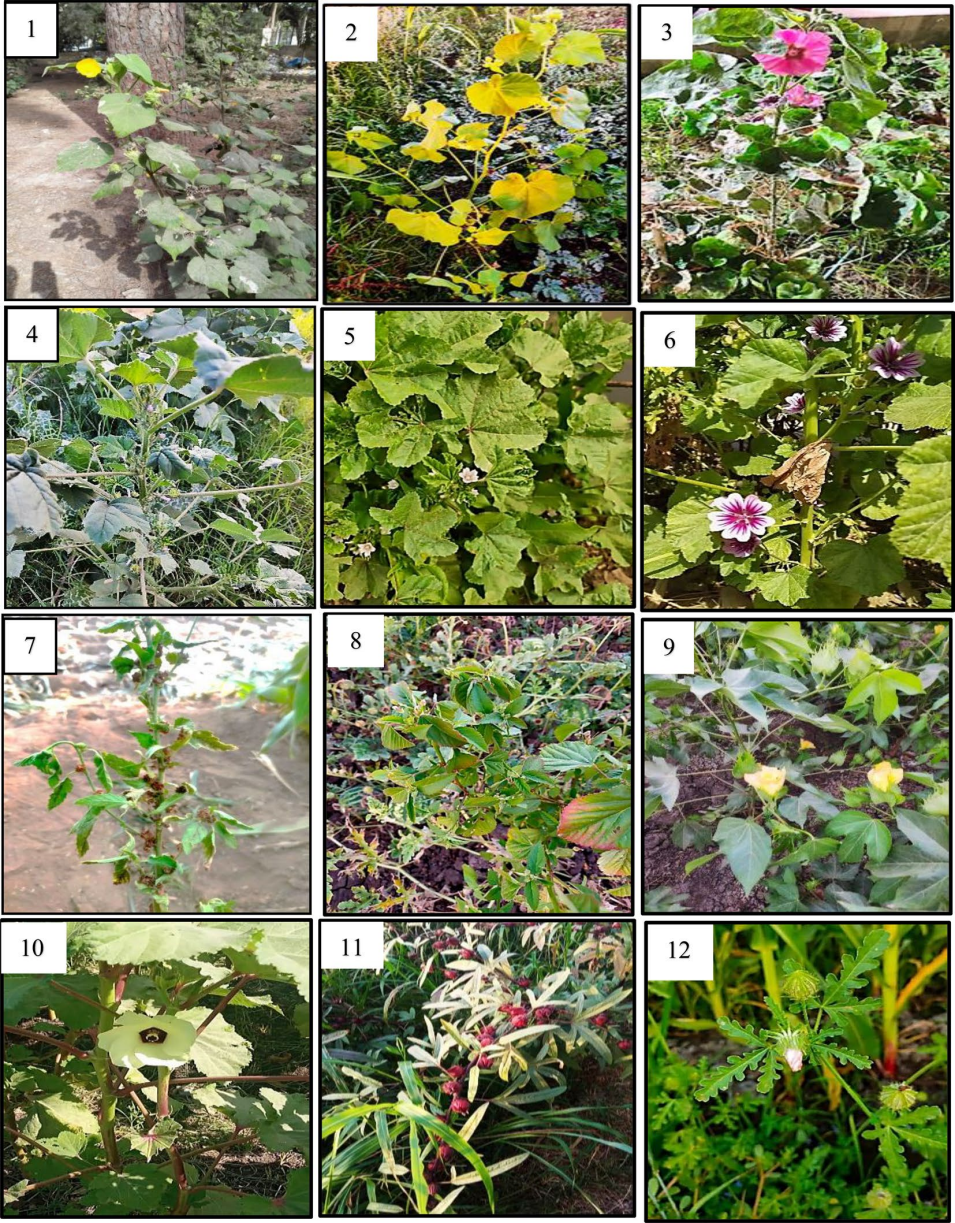
Photos for some studied species of Malvaceae: (**1**) Abutilon grandifolium, (**2**) Abutilon theophrasti, (**3**) Alcea rosea, (**4**) Malva multiflora, (**5**) Malva nicaeensis and (**6**) Malva sylvestris, (**7**) Malvastrum coromandelianum, (**8**) Sida alba, (**9**) Gossypium barbadense, (**10**) Abelomochus esculentus, (**11**). Hibiscus sabdariffa, and (**12**). Hibiscus trionum

Within the studied species of Malvaceae, growth forms of the plants ranged between annual, perennial, herbs, and shrubs (Table 3; Fig. 1). Annual herbs are in most studied species, and perennial shrubs are in *Abutilon grandifolium*, *A*. *pannosum*, and *A*. *theophrasti*.

### Stem

The stem is solid and cylindrical in all studied species. It is usually erect but erect to decumbent in *Malva neglecta*,* M*. *nicaeensis*, *M*. *parviflora*, and *M*. *sylvestris*. The stem is branched in most species but unbranched in *Alcea rosea* and *Abelomochus esculentus*. The texture of the stem is usually pubescent, but tomentose in *Abutilon grandifolium* and *A*. *pannosum* and glabrous in *Gossypium barbadense* only. The stem color is green in all studied species but red in *Hibiscus sabdariffa*. The stem surface is smooth in *Abutilon pannosum*,* A*. *theophrasti*,* Malva parviflora*, *M*. *sylvestris*, *Sida alba*, and *Gossypium barbadense*, while rough in the remainders. Internode length ranged between very short (1.7–2.5 cm) in *Malva neglecta*, long (5–7 cm) in *Abutilon theophrasti*,* Gossypium barbadense*, and *Abelomochus esculentus*, very long (more than 7 cm) in *Hibiscus trionum* only, and short (3–4.5 cm) in the rest.

### Stipule

The leaves are stipulate in all studied species. The stipule is membranous in *Malva neglecta*,* M*. *nicaeensis*,* M*. *parviflora*, and *M*. *sylvestris*, whereas it is normal in the rest of the species. Stipule shape ranged from lanceolate in *Malvastrum coromandelianum* and *Gossypium barbadense*, triangular in *Abutilon grandifolium*,* A*. *pannosum*, *A*. *theophrasti*,* Alcea rosea*,* Malva multiflora*,* M. neglecta* and *M*. *parviflora*, ovate in *Malva nicaeensis* and *M*. *sylvestris*, and linear in *Sida alba*,* Abelomochus esculentus*,* Hibiscus sabdariffa*, and *H*. *trionum*. The apex of the stipule is acute in most species but acuminate in *Abutilon grandifolium*,* Malva nicaeensis*, *Malvastrum coromandelianum*,* Gossypium barbadense*,* Abelomochus esculentus*,* Hibiscus sabdariffa*, and *H*. *trionum*. The surface of the stipule is tomentose in *Abutilon grandifolium*,* A*. *pannosum*, and *A*. *theophrasti*, glabrous in *Gossypium barbadense* only, and pubescent in the remainders. Dark veins in the stipule are present in *Abutilon grandifolium*,* Malva multiflora*,* M. neglecta*,* M*. *nicaeensis*,* M*. *parviflora*,* M*. *sylvestris*, and *Gossypium barbadense* and absent in the rest.

Stipule length is short (3.2–7 mm) in most studied species, while long (9–12 mm) in *Sida alba*, and very long (more than 12 mm) in *Abutilon grandifolium*,* Gossypium barbadense*, and *Abelomochus esculentus*. The width of stipule ranged from narrow (0.3–2.3 mm) as in most studied species, to wide (3.5–5.2 mm) as in *Alcea rosea*,* Malva multiflora*,* M. nicaeensis*, and *Gossypium barbadense*.

**Table 3:** Morphological characters of growth form, stem, and stipule

Species	Characters													
	Growth form		Stem					Stipule						
	Duration:1-annual/2-perennial	Habit: 1-herb/2-shrub.	Nature: 1-erect/2-erect to decumbent.	Branching: 1-branched/2-unbranched.	Texture: 1-pubescent/2-tomentose/3-glabrescent.	Color: 1-green/2-red.	Internode length cm: 1-very short 1.7-2.5/2-short 3-4.5/3-long 5-7/4-very long more than 7.	Stipule: 1-normal/2-membranous.	Shape: 1-lanceolate/2-triangular/3-ovate/4-linear.	Apex: 1-acute /2-acuminate.	Texture: 1-pubescent/2-tomentose /3-glabrous.	Dark veins: 1-present/2-absent.	Length mm: 1-short 3.2-7/2-long 9-12 /3-very long more than 12.	Width mm: 1- narrow 0.3-2.3/2- wide 3.5-5.2.
*Abutilon grandifolium*	2	2	1	1	2	1	2	1	2	2	2	1	3	1
*Abutilon pannosum*	2	2	1	1	2	1	2	1	2	1	2	2	1	1
*Abutilon theophrasti*	2	2	1	1	1	1	3	1	2	1	2	2	1	1
*Alcea rosea*	1	1	1	2	1	1	2	1	2	1	1	2	1	2
*Malva multiflora*	1	1	1	1	1	1	2	1	2	1	1	1	1	2
*Malva neglecta*	1	1	2	1	1	1	1	2	2	1	1	1	1	1
*Malva nicaeensis*	1	1	2	1	1	1	2	2	3	2	1	1	1	2
*Malva parviflora*	1	1	2	1	1	1	2	2	2	1	1	1	1	1
*Malva sylvestris*	1	1	2	1	1	1	2	2	3	1	1	1	1	1
*Malvastrum coromandelianum*	1	1	1	1	1	1	2	1	1	2	1	2	1	1
*Sida alba*	1	1	1	1	1	1	2	1	4	1	1	2	2	1
*Gossypium barbadense*	1	1	1	1	3	1	3	1	1	2	3	1	3	2
*Abelomochus esculentus*	1	1	1	2	1	1	3	1	4	2	1	2	3	1
*Hibiscus sabdariffa*	1	1	1	1	1	2	2	1	4	2	1	2	1	1
*Hibiscus trionum*	1	1	1	1	1	1	4	1	4	2	1	2	1	1

**Fig. 2:**
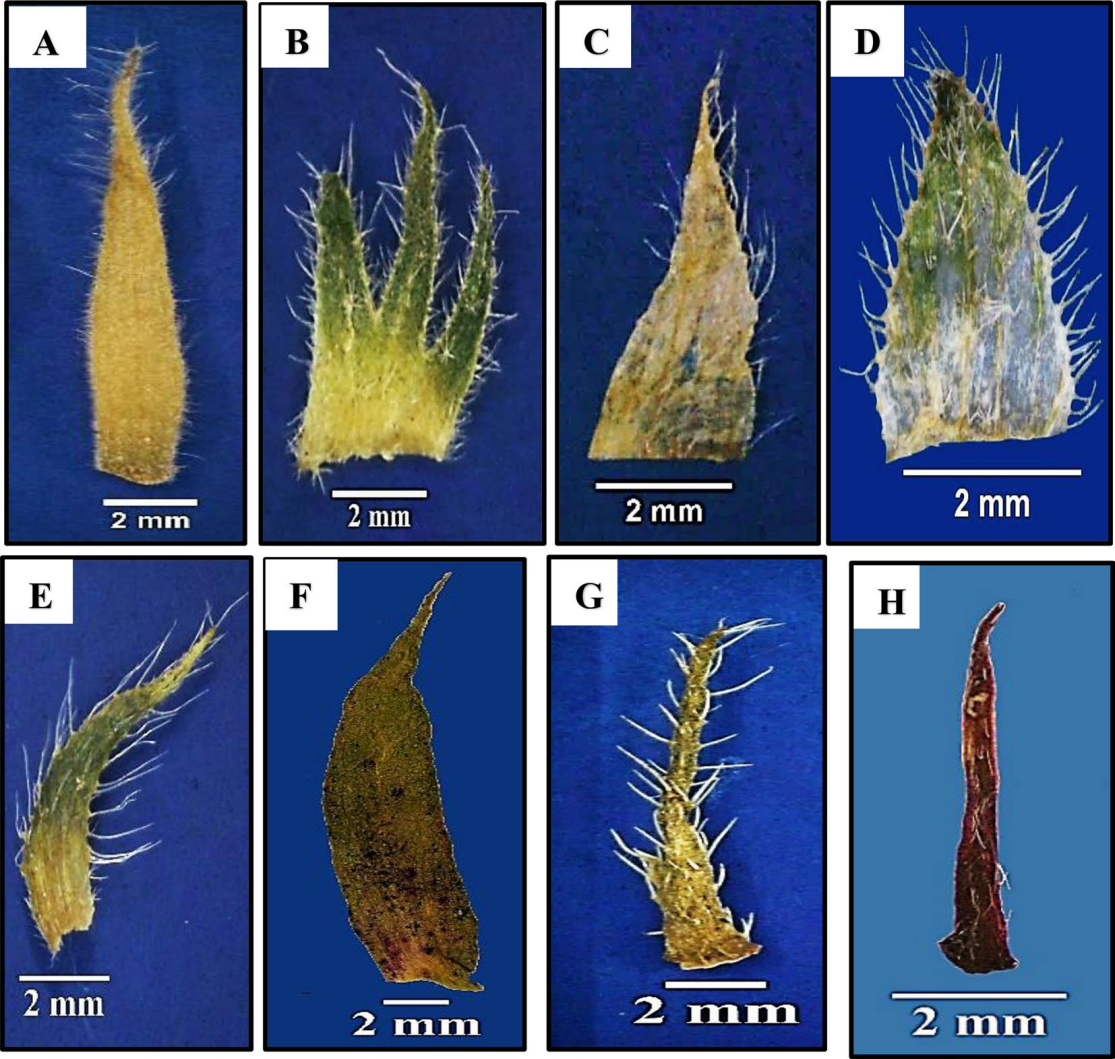
Different shapes of stipules for studied species of Malvaceae as revealed by light microscope: (**A**) *Abutilon grandifolium*, (**B**) *Alcea rosea*, (**C**) *Malva parviflora*, (**D**) *Malva sylvestries*, (**E**) *Malvastrum coromandelianum*, (**F**) *Gossypium barbadense*, (**G**) *Abelomochus esculentus*, and (**H**) *Hibiscus sabdariffa*

### Leaf

**Fig. 3:**
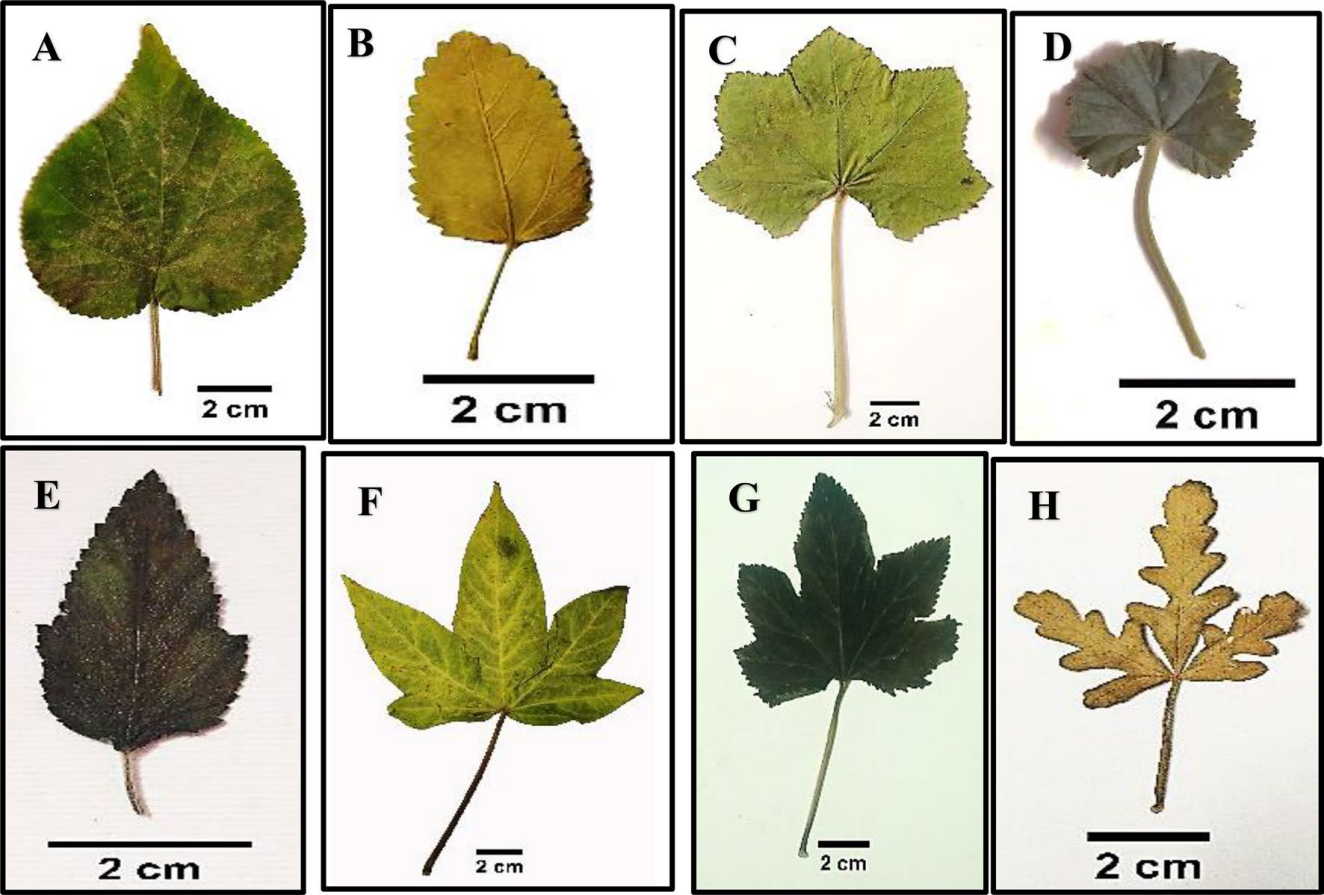
Morphological characters of leaves for studied species of Malvaceae as revealed by light microscope: (**A**) *Abutilon grandifolium*, (**B**) *Sida alba*, (**C**) *Malva multiflora*, (**D**) *Malva neglecta*, (**E**) *Malvastrum coromandelianum*, (**F**) *Gossypium barbadense*, (**G**) *Abelomochus esculentus*, and (**H**) *Hibiscus trionum*

The leaves of the studied species show great variations in shape, texture, margin, apex, and measurements, as seen in Table 4; Fig. 3. Leaves are usually petiolate; the petiole surface is tomentose in *Abutilon grandifolium*, *A*. *pannosum*, and *Malvastrum coromandelianum*, while glabrous in *Gossypium barbadense* only and pubescent in the remainders. The blade composition is entire in *Abutilon grandifolium*,* A*. *pannosum*,* A*. *theophrasti*,* Alcea rosea*, and *Sida alba*, while lobed in the remainders. The lobed leaves are varied in shape, ranging between palmatifid in most studied species, palmatisect in *Hibiscus sabdariffa* and *H*. *trionum*, palmatipartite in *Gossypium barbadense* and *Abelomochus esculentus*, and pinnatifid in *Malvastrum coromandelianum*.

Leaf blade segment was found to be lobed in *Hibiscus trionum* but entire in *Malva multiflora*,* M. neglecta*,* M*. *nicaeensis*, *M*. *parviflora*,* M*. *sylvestris*,* Malvastrum coromandelianum*, *Gossypium barbadense*,* Abelomochus esculentus* and *Hibiscus sabdariffa*. Venation of the blade is mostly palmately nerved, except in *Malvastrum coromandelianum* and *Sida alba* it is pinnately nerved. The apex is acute in *Abutilon pannosum*,* Malva multiflora*,* Malvastrum coromandelianum*,* Sida alba*,* Abelomochus esculentus*, and *Hibiscus sabdariffa*, obtuse in *Alcea rosea*,* Malva neglecta*,* M*. *nicaeensis*,* M*. *parviflora*,* M*. *sylvestris*, and *Hibiscus trionum*, and acuminate in *Abutilon grandifolium*,* A*. *theophrasti* and *Gossypium barbadense*,.

Also, the margin recorded five types: serrate in *Malvastrum coromandelianum* and *Hibiscus sabdariffa*, lobate in *Hibiscus trionum*, entire in *Gossypium barbadense*, and dentate margin in the remainders. Leaf texture is pubescent in most studied species, but glabrescent in *Gossypium barbadense* and tomentose in *Abutilon grandifolium* and *A*. *pannosum*.

**Table 4:** Morphological characters of leaf

Species	Characters										
	Petiole		Blade								
	Surface: 1-pubescent /2-tomentose/3-glabrous.	Lengthcm: 1-short 1.4-4.5/2-long 6-8.6/3-very long 10-14.2.	Composition: 1-entire/2-lobed.	Shape: 1-entire/2-palmatifid/3-palmatisect/4-palmatipartite/ 5-pinnatifid.	Segment: 1-lobed/2-entire/3-absent.	Venation: 1-pinnately nerved/2-palmately nerved.	Apex: 1-acute /2-acuminate /3-obtuse.	Margin: 1-dentate/2-lobate/3-serrate/4-entire.	Texture: 1-pubescent/2-tomentose/3-glabrescent.	Length cm: 1-short 1.3-4.5/2-long 5-6.5/3-very long 8-13.4.	Width cm:1-1.7-3.2/2-4-7/3-more than 7.
*Abutilon grandifolium*	2	1	1	1	3	2	2	1	2	3	3
*Abutilon pannosum*	2	1	1	1	3	2	1	1	2	2	2
*Abutilon theophrasti*	1	2	1	1	3	2	2	1	1	3	3
*Alcea rosea*	1	2	1	1	3	2	3	1	1	3	3
*Malva multiflora*	1	2	2	2	2	2	1	1	1	3	3
*Malva neglecta*	1	1	2	2	2	2	3	1	1	1	1
*Malva nicaeensis*	1	3	2	2	2	2	3	1	1	2	2
*Malva parviflora*	1	3	2	2	2	2	3	1	1	2	2
*Malva sylvestris*	1	2	2	2	2	2	3	1	1	1	1
*Malvastrum coromandelianum*	2	1	2	5	2	1	1	3	1	2	1
*Sida alba*	1	1	1	1	3	1	1	1	1	1	2
*Gossypium barbadense*	3	2	2	4	2	2	2	4	3	3	3
*Abelomochus esculentus*	1	3	2	4	2	2	1	1	1	3	3
*Hibiscus sabdariffa*	1	1	2	3	2	2	1	3	1	3	3
*Hibiscus trionum*	1	1	2	3	1	2	3	2	1	1	2

### Floral morphology

#### Inflorescence

The flower was found to be solitary in *Gossypium barbadense*,* Abelomochus esculentus*,* Hibiscus sabdariffa*, and *H*. *trionum*, while cymose monochasial in *Abutilon grandifolium*,* A*. *pannosum*, and *A*. *theophrasti* and racemose in the remainder. The flower is always pedicellate, with pedicel length being very short 0.2–0.9 cm in *Malva neglecta*,* Malvastrum coromandelianum*,* Abelomochus esculentus*, and *Hibiscus sabdariffa*, long (2.3–4 cm) in *Abutilon grandifolium*,* Gossypium barbadense*, and *Hibiscus trionum*, while short (1–1.7 cm) in the remainder. The pedicel surface is pubescent in most species but tomentose in *Abutilon grandifolium*,* A*. *pannosum*,* A*. *theophrasti*, and *Malvastrum coromandelianum* and glabrous in *Gossypium barbadense* only, as shown in Table 5.

#### Hypocalyx and calyx

**Fig. 4:**
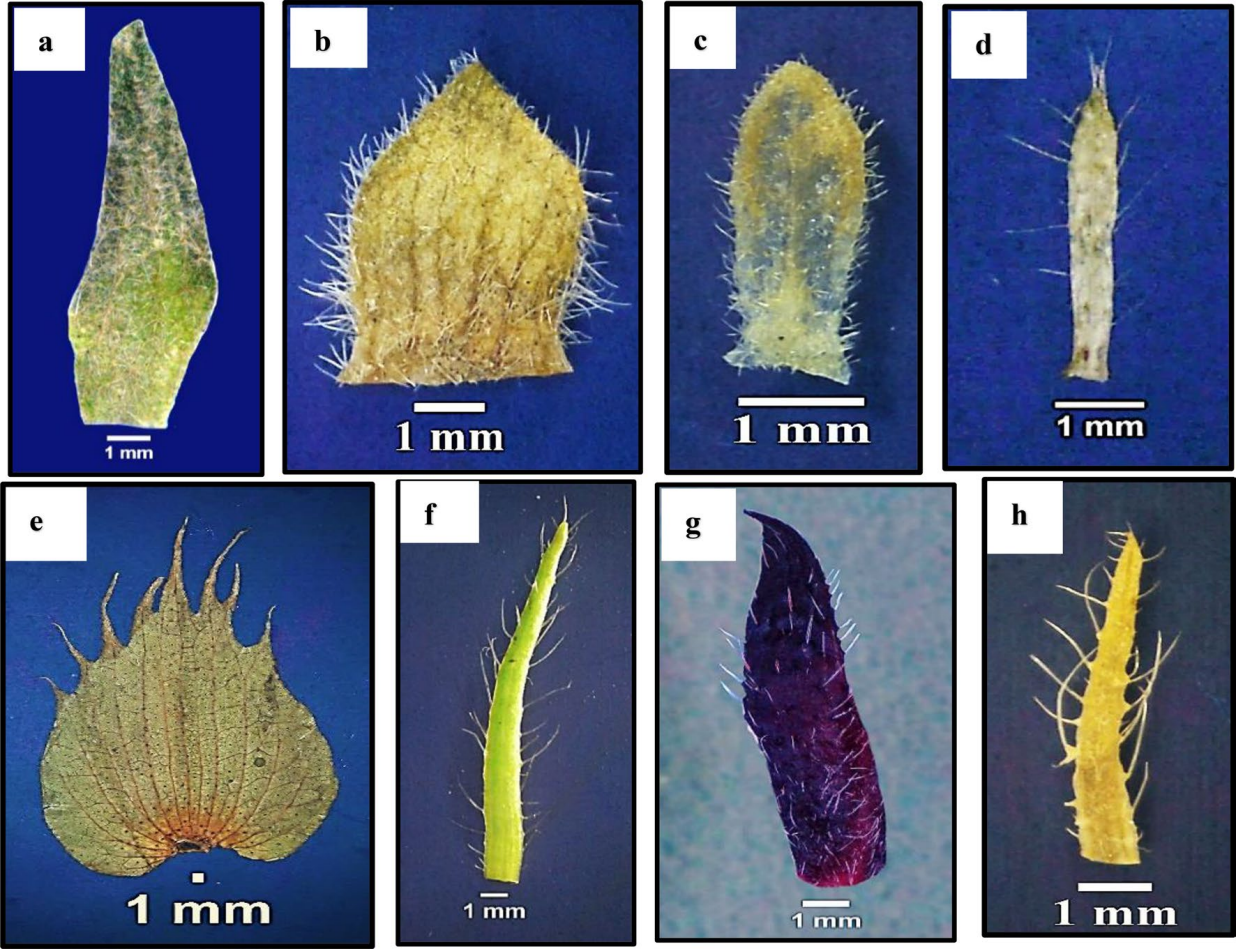
Different shapes of hypocalyx for studied species of Malvaceae as revealed by light microscope: **a**. *Alcea rosea*, **b**. *Malva multiflora*, **c**. *Malva neglecta*, **d**. *Malva parviflora*, **e**. *Gossypium barbadense*, **f**. *Abelomochus esculentus*, **g***Hibiscus sabdariff*a, and h. *Hibiscus trionum*

Hypocalyx is present in most species but absent in *Abutilon grandifolium*,* A*. *pannosum*,* A*. *theophrasti*, and *Sida alba*. The shape of the hypocalyx unit varies between species studied, being linear as in *Malva parviflora*,* Malvastrum coromandelianum*,* Abelomochus esculentus*, and *Hibiscus trionum*, oblong as in *Malva neglecta* and *M*. *sylvestris*, and broad ovate in *Malva multiflora*,* M. nicaeensis*, and *Gossypium barbadense*, while lanceolate in *Alcea rosea* and *Hibiscus sabdariffa*. Apex is mostly acute, except in *Gossypium barbadense*, which is toothed. The hypocalyx surface is glabrous in *Gossypium barbadense* only and pubescent in the rest of the species. The number of hypocalyx units ranged three in most studied species, seven to ten in *Alcea rosea* and *Hibiscus sabdariffa*, and more than 10 in *Abelomochus esculentus* and *Hibiscus trionum*, as shown in Table 5; Fig. 4.

The length of the hypocalyx unit is very short (2.6–6 mm) in most studied species, short (8.7–13.1 mm) in *Alcea rosea*,* Abelomochus esculentus*,* Hibiscus sabdariffa*, and *H*. *trionum*, or long (more than 13.1 mm) in *Gossypium barbadense* only. Hypocalyx unit width ranged between (0.3–1 mm) in *Malva neglecta*,* M*. *parviflora*,* M*. *sylvestris*, *Malvastrum coromandelianum* and *Hibiscus trionum*, (1.5–2 mm) in *Abelomochus esculentus* and *Hibiscus sabdariffa*, (2.3–5 mm) in *Alcea rosea*,* Malva multiflora* and *M. nicaeensis* and more than 5 mm in *Gossypium barbadense* only.

**Fig. 5:**
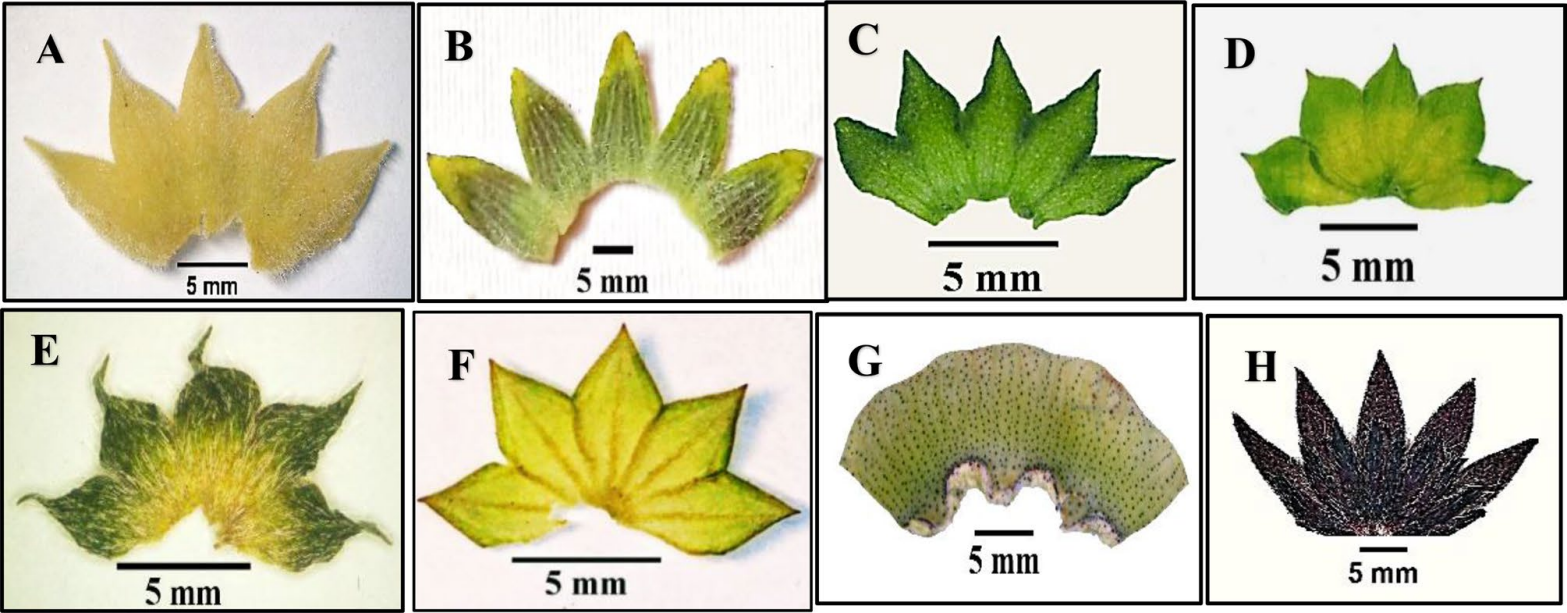
Shapes of calyx for studied species of Malvaceae as revealed by light microscope: (**A**) Abutilon pannosum, (**B**) Alcea rosea, (**C**) Malva neglecta, (**D**) Malva parviflora, (**E**) Malvastrum coromandelianum, (**F**) Sida alba, (**G**) Gossypium barbadense, and (**H**) Hibiscus sabdariffa

Calyx with five gamosepalous sepals. Sepals with green color found in *Malva neglecta*,* M*. *sylvestris*, and *Abelomochus esculentus*, red in *Hibiscus sabdariffa* only, and yellowish-green in the rest. Sepal length, short (7–12.3 mm) in *Abutilon grandifolium*,* A*. *pannosum*,* A*. *theophrasti*,* Malva sylvestris* and *Gossypium barbadense*, long (more than 12.3 mm) in *Alcea rosea*,* Abelomochus esculentus*,* Hibiscus sabdariffa* and *H*. *trionum*, while very short 4.6–6.6 mm in the remainders. The sepal apex is mostly acuminate but acute in *Abutilon theophrasti*,* Alcea rosea*,* Sida alba*,* Abelomochus esculentus*,* Hibiscus sabdariffa*, and *H*. *trionum*, while obtuse in *Gossypium barbadense* only. Sepals are pubescent in most studied species, glabrous in *Gossypium barbadense* only, and tomentose in *Abutilon pannosum*, as seen in Table 5; Fig. 5.

**Table 5:** Morphological characters of flower, pedicel, and hypocalyx

Species	Characters														
	Flower: 1-solitary/2-racemose/3- cyme monochasial.	Pedicel		Hypocalyx							Calyex				
		Surface: 1-pubescent/2-tomentose/3-glabrous.	Length cm: 1-very short 0.2-0.9/2-short 1-1.7 /3-long 2.3-4.	Hypocalyx 1-present/2-absent.	Shape: 1-linear/2-oblong/3-broad ovate / 4-lanceolate/ 5-absent.	Apex: 1-acute/ 2-toothed/ 3-absent.	Surface: 1-pubescent/ 2-glabrous/ 3-absent.	Number: 1- three /2-seven to ten /3-more than 10/ 4-absent.	Length mm: 1- very short 2.6-6/ 2-short 8.7-13.1 /3-long more than 13.1/4- absent.	Width mm: 1- 0.3-1/2- 1.5-2 / 3- 2.3-5/4- more than 5/ 5-absent.	Color: 1-green/2-yellowish green /3- red.	Apex: 1-acute /2-acuminate /3-obtuse.	Surface: 1-pubescent /2-tomentose /3-glabrous.	Tube length mm: 1-short 0.1-0.4/2-long 0.5-0.7/3- very long more than 0.7.	Sepal length mm: 1- very short 4.6- 6.6/ 2-short 7- 12.3/3- long more than 12.3.
*Abutilon grandifolium*	3	2	3	2	5	3	3	4	4	5	2	2	1	1	2
*Abutilon pannosum*	3	2	2	2	5	3	3	4	4	5	2	2	2	2	2
*Abutilon theophrasti*	3	2	2	2	5	3	3	4	4	5	2	1	1	1	2
*Alcea rosea*	2	1	2	1	4	1	1	2	4	5	2	1	1	1	3
*Malva multiflora*	2	1	2	1	3	1	1	1	1	3	2	2	1	2	1
*Malva neglecta*	2	1	1	1	2	1	1	1	1	1	1	2	1	2	1
*Malva nicaeensis*	2	1	2	1	3	1	1	1	1	3	2	2	1	2	1
*Malva parviflora*	2	1	2	1	1	1	1	1	1	1	2	2	1	2	1
*Malva sylvestris*	2	1	2	1	2	1	1	1	1	1	1	2	1	1	2
*Malvastrum coromandelianum*	2	2	1	1	1	1	1	1	1	1	2	2	1	2	1
*Sida alba*	2	1	2	2	5	3	3	4	4	5	2	1	1	2	1
*Gossypium barbadense*	1	3	3	1	3	2	2	1	3	4	2	3	3	3	2
*Abelomochus esculentus*	1	1	1	1	1	1	1	3	2	2	1	1	1	3	3
*Hibiscus sabdariffa*	1	1	1	1	4	1	1	2	2	2	3	1	1	2	3
*Hibiscus trionum*	1	1	3	1	1	1	1	3	2	1	2	1	1	2	3

### Corolla

Corolla is generally with five, free and showy petals. Petals color is yellow in most studied specis, pale-violet in *Malva M. multiflora*,* M*. *neglecta*,* M*. *nicaeensis*,* M*. *parviflora* and *M*. *sylvestris*, off-white in *Sida alba* and *Hibiscus trionum*, pink in *Alcea rosea* and red in *Hibiscus sabdariffa* only. Petals with monomorphic color found in *Abutilon grandifolium*,* A*. *theophrasti*,* Alcea rosea*,* Malvastrum coromandelianum*,* Sida alba*, and *Hibiscus sabdariffa*, while dimorphic in the remainer.

**Fig. 6:**
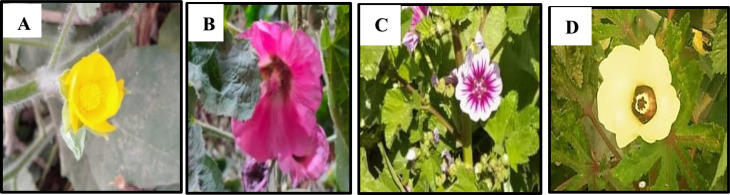
Corolla for some studied species of Malvaceae: (**A**) *Abutilon grandifolium*, (**B**) *Alcea rosea*, (**C**) *Malva sylvestries*, and (**D**) *Abelomochus esculentus*

**Fig. 7:**
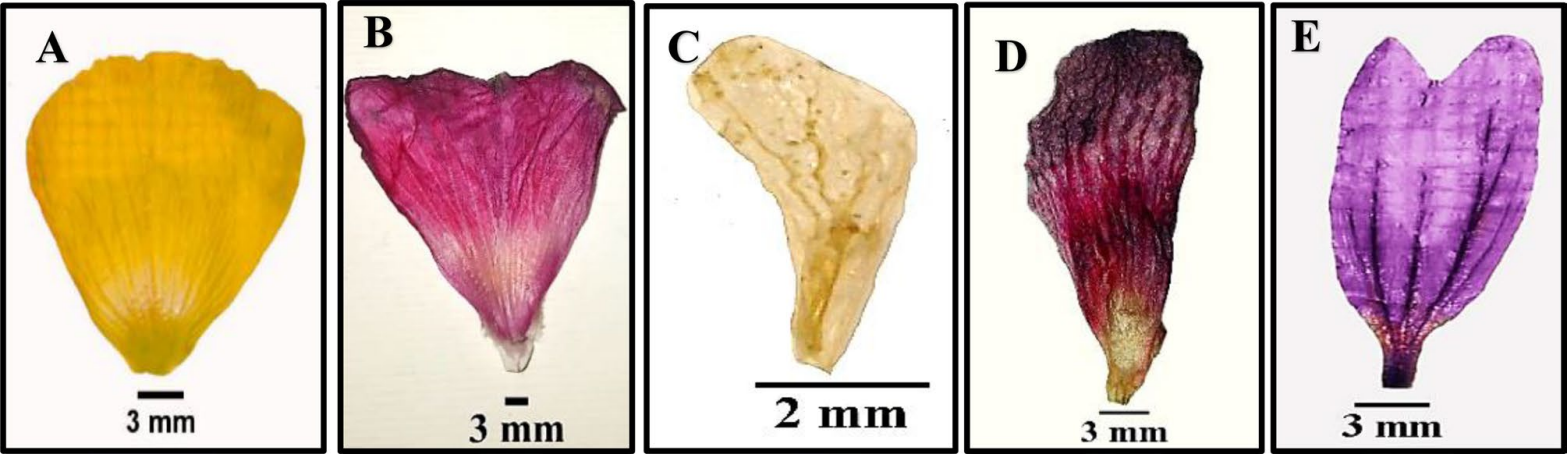
Morphological characters of petal for studied species of Malvaceae as revealed by light microscope: (**A**) *Abutilon grandifolium*, (**B**) *Alcea rosea*, (**C**) *Sida alba*, (**D**) *Hibiscus sabdariffa*, and (**E**) *Malva sylvestries*

Petals with purple spots color as in *Abutilon pannosum*, *Gossypium barbadense*,* Abelomochus esculentus* and *Hibiscus trionum*. Petals usually have dark veins, but absent in *Abutilon grandifolium*, *A*. *theophrasti*, and *Alcea rosea*. Clawed petals are detected in *Abutilon pannosum*, *Malva multiflora*,* M. parviflora*, *Sida alba*,* Gossypium barbadense*,* Abelomochus esculentus* and *Hibiscus sabdariffa*. The apex of petal is emarginate in *Malva multiflora*,* M*. *neglecta*,* M*. *nicaeensis*,* M*. *parviflora*,* M*. *sylvestris*, and *Malvastrum coromandelianum*, but not emarginate in the rest as shown in (Table 6; Figs. 6 and 7).

### Androecium

Androecium being with numerous stamens, filaments united to form a monadelphous, epipetalous staminal tube, contains unilocular anthers. The surface of the staminal tube is glabrous in most studied species and hairy in *Abutilon grandifolium*,* A*. *pannosum*, and *Hibiscus trionum*. Staminal tube color differs among studied species; it is pale yellow in most studied species, yellow color in *Abutilon grandifolium*,* A*. *theophrasti*,* Malvastrum coromandelianum* and *Sida alba*, purple to yellow in *Abutilon pannosum*, purple in *Malva sylvestris*,* Hibiscus sabdariffa* and *H. trionum*, and pink in *Alcea rosea*.

**Fig. 8:**
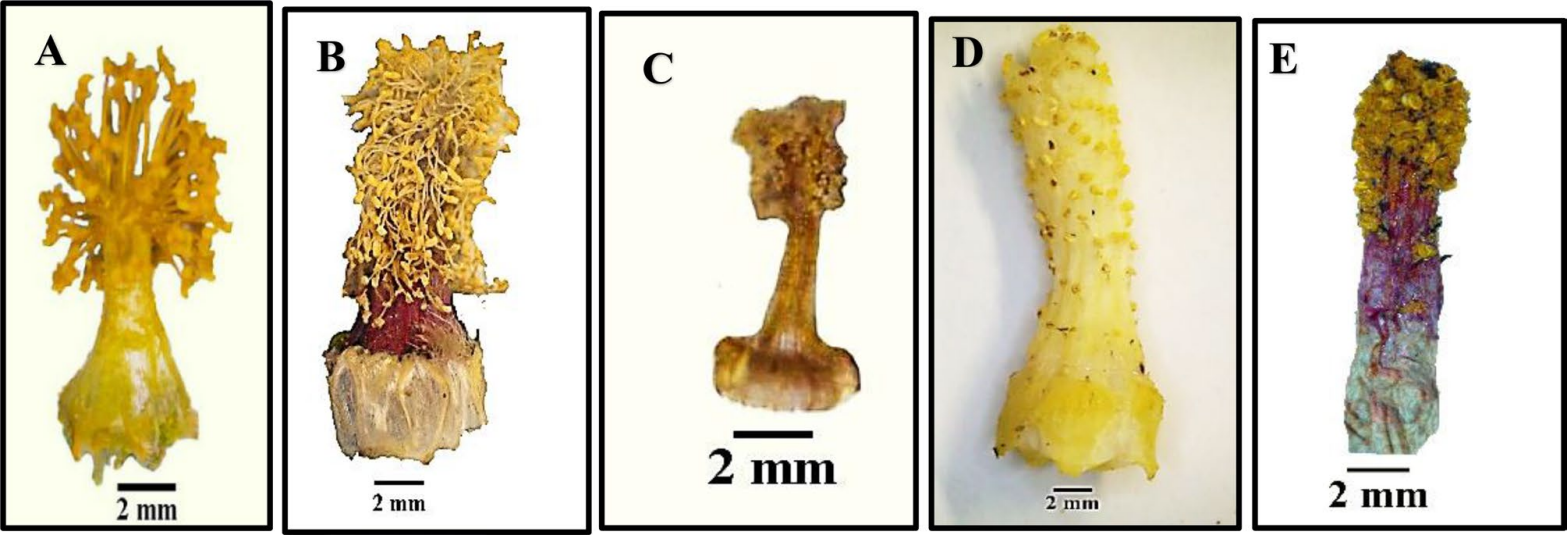
Morphological characters of staminal tube for studied species of Malvaceae as revealed by light microscope: **A**. *Abutilon grandifolium*, **B**. *Alcea rosea*, **C**. *Malva multiflora*, **D**. *Abelomochus esculentus*, and E. *Hibiscus sabdariffa*

Anthers usually found crowded at the tip of staminal tube or being spread along the tube as in *Gossypium barbadense*,* Abelomochus esculentus*,* Hibiscus sabdariffa* and *H*. *trionum*. The staminal tube varied in length among studied species, long staminal tube (1.6–3 cm) as in *Alcea rosea*,* Gossypium barbadense* and *Abelomochus esculentus*, short (1–1.2 cm) in *Abutilon grandifolium*,* A*. *pannosum*,* Hibiscus sabdariffa* and *H*. *trionum*, while very short (0.4–0.8 cm) in the rest, as shown in (Table 6; Fig. 8).

### Gynoecium

**Fig. 9:**
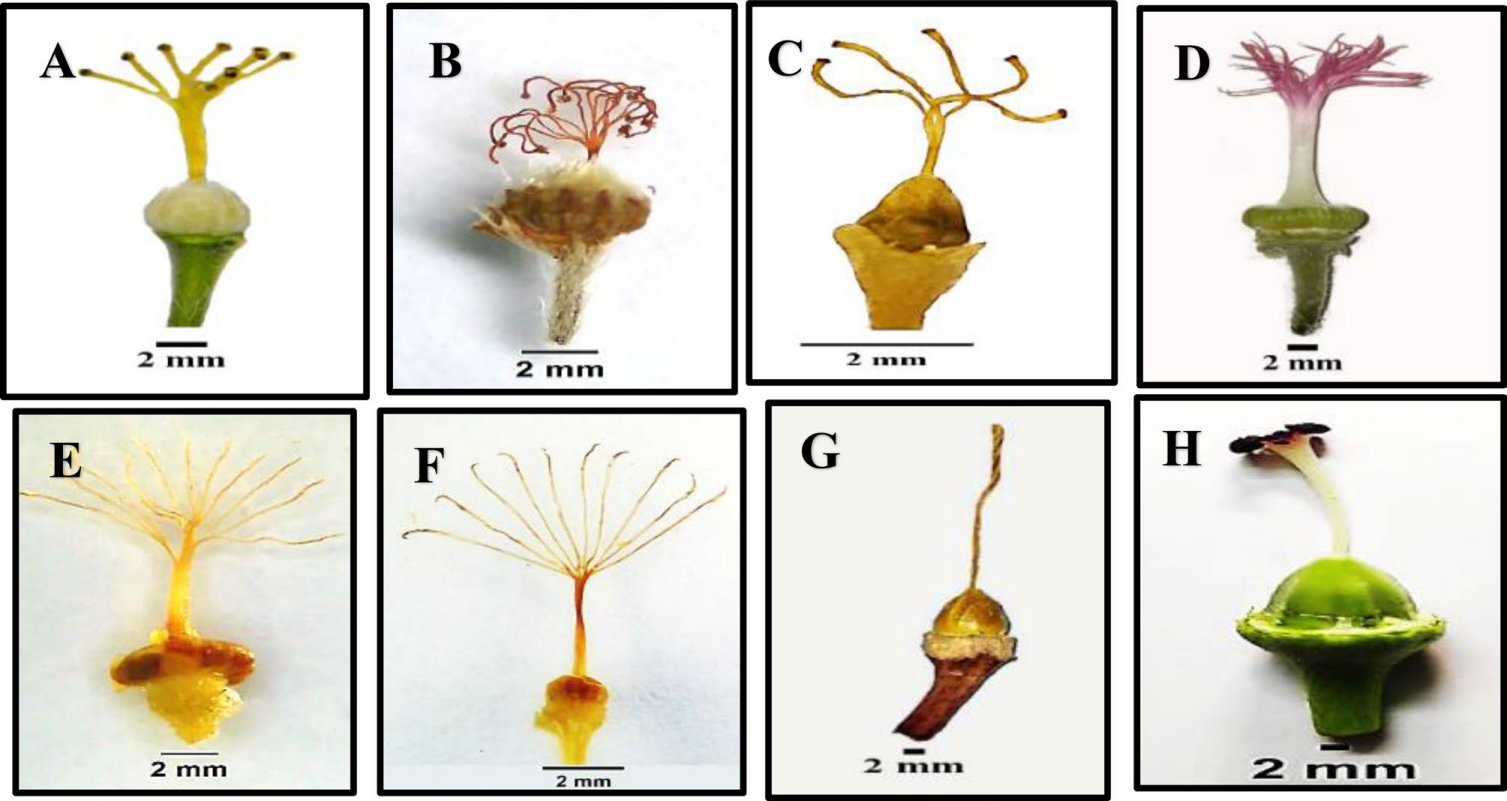
Gynoecium for studied species of Malvaceae as revealed by light microscope: **A**. *Abutilon grandifolium*, **B**. Malvastrum *coromandelianum*, **C**. *Sida alba*, **D**. *Alcea rosea*, **E**. *Malva neglecta*, **F**. *Malva sylvestries*, **G**. *Gossypium barbadense*, and H. *Abelomochus esculentus*

The gynoecium is syncarpous, ovary superior, penta, or multilocular with axile placentation as shown in (Table 6; Fig. 9). The surface of the ovary is pubescent in five studied species: *Alcea rosea*,* Malvastrum coromandelianum*,* Abelomochus esculentus*,* Hibiscus sabdariffa*, and *H*. *trionum*, tomentose in *Abutilon grandifolium*,* A*. *pannosum*, and *A*. *theophrasti*, while glabrous in the rest.

Ovary shape ranged between sub-globous as in *Abutilon pannosum*,* A*. *theophrasti*, and *Malva multiflora*, ovate as in *Abutilon grandifolium*,* Sida alba*,* Gossypium barbadense*,* Abelomochus esculentus*,* Hibiscus sabdariffa* and *H*. *trionum*, while discoid in the remainders. Ovary length was very short (0.7–1.5 mm) as in most studied species, short (2.5–3 mm) as in *Abutilon grandifolium*,* A*. *pannosum*,* A*. *theophrasti*,* Alcea rosea*, and *Malvastrum coromandelianum*, or long (5–10 mm) in *Gossypium barbadense*,* Abelomochus esculentus*,* Hibiscus sabdariffa* and *H*. *trionum*. Ovary width is (3–4 mm) in *Abutilon grandifolium*,* A*. *pannosum*, and *A*. *theophrasti*, while (5–8 mm) in *Alcea rosea*,* Gossypium barbadense*,* Abelomochus esculentus*,* Hibiscus sabdariffa* and *H*. *trionum*, and (1.4–2.5 mm) in the rest,

Style length varies within species; very short (0.8–2 mm) in *Abutilon theophrasti*,* Malva parviflora and Malvastrum coromandelianum*, short (2.3–4.8 mm) in *Malva multiflora*,* M*. *neglecta*,* M*. *nicaeensis*,* M*. *sylvestris* and *Sida alba*, long (6–10 mm) in *Abutilon grandifolium*,* A*. *pannosum*,* Hibiscus sabdariffa*, and *H*. *trionum* and very long more than 10 mm in *Alcea rosea*,* Gossypium barbadense*, and *Abelomochus esculentus*. Also, there is variation in stigma type and number; the stigma type is slightly clavate in *Gossypium barbadense* only, discoid as in *Abelomochus esculentus*,* Hibiscus sabdariffa*, and *H*. *trionum*, and terete in the rest of species. The number of stigma was found to be three as in *Gossypium barbadense* only, five stigma as in *Sida alba*,* Abelomochus esculentus*,* Hibiscus sabdariffa*, and *H*. *trionum*, from 8 to 10 in *Abutilon grandifolium*,* Malva multiflora*,* M. nicaeensis*,* M*. *parviflora*, and *M*. *sylvestris*, more than 10 in *A*. *pannosum*,* A*. *theophrasti*,* Alcea rosea*,* Malva neglecta*, and *Malvastrum coromandelianum*.

**Table 6:** Macro–morphological characters of the corolla, staminal tube, and gynoecium

Species	Characters																		
	Corolla								Staminal tube				Ovary				Style Length mm:1-very short 0.8-2 /2-short 2.3-4.8/3-long 6-10/ 4-very long more than 10.	Stigma	
	1-yellow/2-pale violet/3-off white/4-pink /5- red.	1- monomorphic/2-dimorphic.	Purple spot: 1-present/2-absent.	Apex: 1-emarginate/2-not emarginate.	Dark veins: 1-present/2-absent.	Claw: 1-clawed/2-not clawed.	Length cm: 1- short 0.4-1.8 /2-long 2.2-3/3-very long 3.5-5.3.	Widthcm: 1- 0.2-0.6/2- 0.9-1.7/3-3-3.5.	Surface: 1-hairy/2-glabrous.	Color:1-yellow/2-purple to yellow/3- pale yellow/ 4-purple/5-pink	Anther: 1-crowded at tip/2-spread.	Length cm: 1-very short 0.4-0.8/2-short 1-1.2/3-long 1.6-3.	Surface: 1-tomentose/2-pubescent/3-glabrous.	Shape: 1-discoid/2-sub-globose/3-ovate.	Length mm: 1-very short 0.7-1.5/ 2-short 2.5-3/ 3-long 5-10.	Width mm: 1- 1.4-2.5/2- 3-4/ 3- 5-8.		Type:1-terete/2-discoid/3-slightly clavate.	Number:1-three /2- five /3- 8-10 /4- more than 10.
*Abutilon grandifolium*	1	1	2	2	2	2	1	2	1	1	1	2	1	3	2	2	3	1	3
*Abutilon pannosum*	1	2	1	2	1	1	1	2	1	2	1	2	1	2	2	2	3	1	4
*Abutilon theophrasti*	1	1	2	2	2	2	1	1	2	1	1	1	1	2	2	2	1	1	4
*Alcea rosea*	4	1	2	2	2	2	3	3	2	5	1	3	2	1	2	3	4	1	4
*Malva multiflora*	2	2	2	1	1	1	1	1	2	3	1	1	3	2	1	1	2	1	3
*Malva neglecta*	2	2	2	1	1	2	1	1	2	3	1	1	3	1	1	1	2	1	4
*Malva nicaeensis*	2	2	2	1	1	2	1	1	2	3	1	1	3	1	1	1	2	1	3
*Malva parviflora*	2	2	2	1	1	1	1	1	2	3	1	1	3	1	1	1	1	1	3
*Malva sylvestris*	2	2	2	1	1	2	2	1	2	4	1	1	3	1	1	1	2	1	3
*Malvastrum coromandelianum*	1	1	2	1	1	2	1	1	2	1	1	1	2	1	2	1	1	1	4
*Sida alba*	3	1	2	2	1	1	1	1	2	1	1	1	3	3	1	1	2	1	2
*Gossypium barbadense*	1	2	1	2	1	1	3	3	2	3	2	3	3	3	3	3	4	3	1
*Abelomochus esculentus*	1	2	1	2	1	1	2	2	2	3	2	3	2	3	3	3	4	2	2
*Hibiscus sabdariffa*	5	1	2	2	1	1	2	2	2	4	2	2	2	3	3	3	3	2	2
*Hibiscus trionum*	3	2	1	2	1	2	2	2	1	4	2	2	2	3	3	3	3	2	2

### Pollen grains morphology

**Fig. 10:**
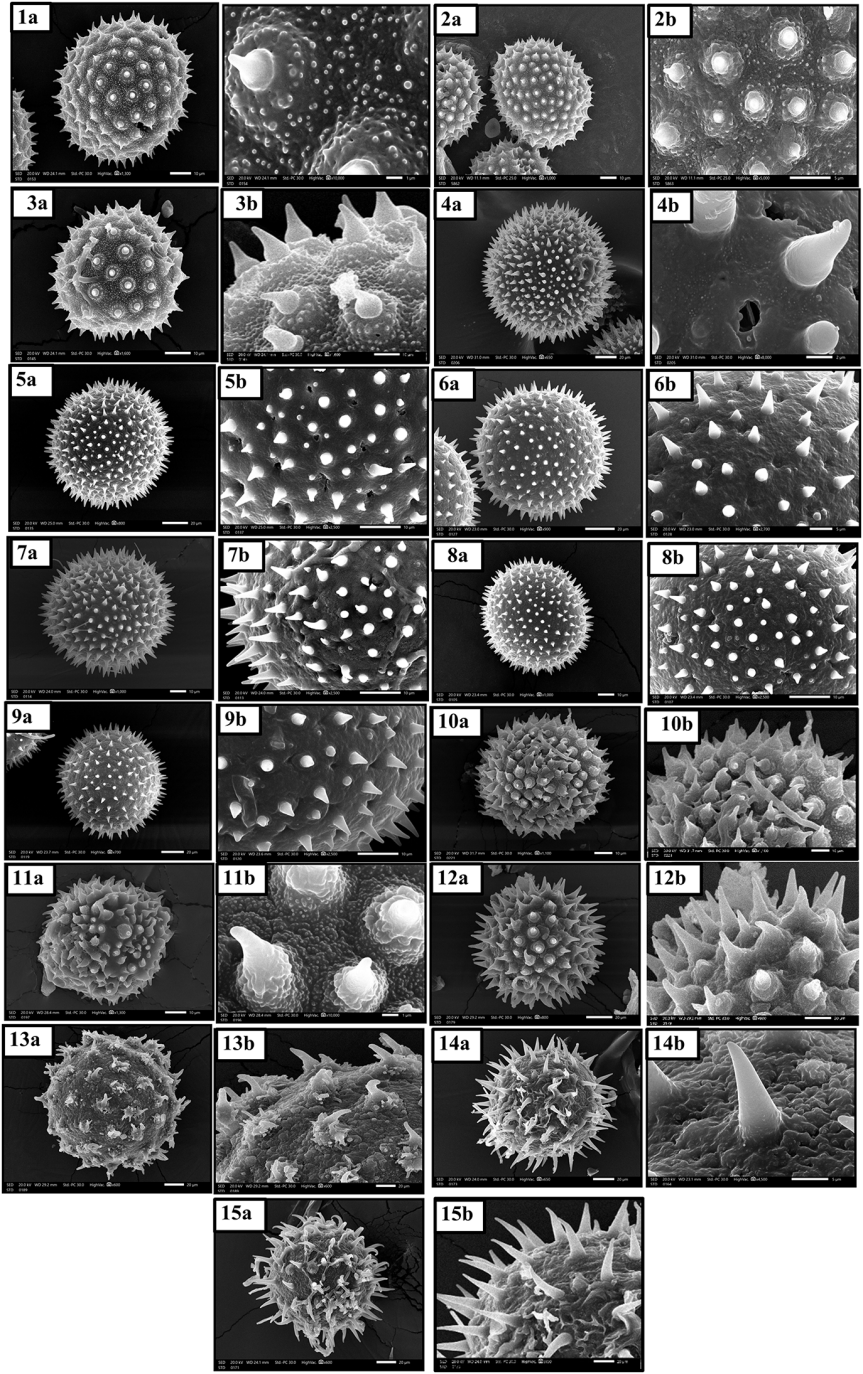
SEM micrographs of pollen grains for studied species of Malvaceae: **1a**-**1b***Abutilon grandifolium*, **2a**-**2b***Abutilon pannosum* and **3a**-**3b***Abutilon theophrasti*, **4a**-**4b***Alcea rosea*, **5a**-**5b***Malva multiflora* and **6a**-**6b***Malva neglecta*. **7a**-**7b***Malva nicaeensis*, **8a**-**8b***Malva parviflora*, **9a**-**9b***Malva sylvestris* and **10a**-**10b***Malvastrum coromandelianum*. **11a**-**11b***Sida alba*, **12a**-**12b***Gossypium barbadense*, **13a**-**13b***Abelomochus esculentus*, **14a**-**14b***Hibiscus sabdariffa*, and **15a**-**15b***Hibiscus trionum*

Pollen grains are variable in shape, size, spine shape, and ornamentation as shown in Table 7; Fig. 10. Pollen grain shapes ranged from prolate-spheroidal as in most studied species, oblate-spheroidal as in *Abutilon grandifolium*,* A*. *pannosum*,* A*. *theophrasti*,* Malva parviflora*, and *Hibiscus sabdariffa*, and spheroidal as in *Malva multiflora*. The size of pollen grains ranged between large (50–99 μm) as in *Abutilon pannosum*,* A*. *theophrasti*,* Malva neglecta*,* M*. *parviflora*, and *Sida alba*, and very large (100–199 μm) in the remainders.

One of the most interesting features of pollen grains in the Malvaceae family is the echination of the exine into definite spines, the spines are variable in their base, length, and shape. Spine length being very long more than 8.8 μm in *Gossypium barbadense*,* Abelomochus esculentus*,* Hibiscus sabdariffa* and *H*. *trionum*, long (5–8.8 μm) in *Malvastrum coromandelianum*, short (3.5–4.5 μm) as in *Abutilon grandifolium*,* A*. *pannosum*,* A*. *theophrasti* and *Sida alba*, or variable in length long (5–8.8 μm + short 3.5–4.5 μm) in *Alcea rosea*,* Malva multiflora*,* M*. *neglecta*,* M*. *nicaeensis*,* M. parviflora*, and *M*. *sylvestris*.

The spine shape is usually conical, while flask-shaped in Abutilon *grandifolium*,* A*. *pannosum*,* A*. *theophrasti*, and *Malvastrum coromandelianum*. Spines are condensed as in *Abutilon grandifolium*,* A*. *pannosum*,* Alcea rosea*,* Malvastrum coromandelianum*,* Sida alba*, and *Gossypium barbadense*, while in the rest of species, spines are spread. Spines are either monomorphic, as in *Abutilon grandifolium*,* A*. *pannosum*,* A*. *theophrasti*,* Malvastrum coromandelianum*,* Sida alba*,* Gossypium barbadense*,* Abelomochus esculentus*,* Hibiscus sabdariffa* and *H*. *trionum*, or dimorphic as in *Alcea rosea*,* Malva multiflora*,* M*. *neglecta*,* M*. *nicaeensis*,* M*. *parviflora*, and *M*. *sylvestris*. Spine base is flat in most studied species, while the spine base is bulbous in *Abutilon grandifolium*,* A*. *pannosum*,* A*. *theophrasti*,* Malvastrum coromandelianum*,* Sida alba*,* Gossypium barbadense*, and *Abelomochus esculentus*. The bulbous surface is granulated in *Abutilon grandifolium*,* A*. *pannosum*,* A*. *theophrasti*, and *Gossypium barbadense*, spiny in Abelomochus *esculentus* only, and verrucated in Malvastrum *coromandelianum* and *Sida alba*. Pollen grain surface ornamentation ranged from; micro-granulate in Alcea *rosea*,* Malva multiflora*, and *M. parviflora*, psilate in *Malva neglecta*,* M*. *nicaeensis* and *M*. *sylvestris*, verrucate in Abelomochus *esculentus*, and granulate in the rest.

**Table 7:** Macro and micromorphological characters of pollen grains

Species	Characters														
	Polar Axis µm		Equatorial diameter µm		PA/ED *100	Pollen shape:1-oblate- spheroidal 88-99 /2-prolate-spheroidal 101-114 /3-spheroidal 100.	Mean size		Spine						
	Mean	Standard ±	Mean	Standard ±				Pollen size µm:1-large 50-99/2-very large 100-199.	Length: 1-short /2-long /3-very long /4-variable.	Shape: 1-conical/2-flask shape.	Density:1-spread/2-condensed.	Type: 1-monomorphic/2-dimorphic.	Base: 1-bulbous/2-flat.	Bulbous surface:1-granulate/2-spiny/3-verrucated /4-absent.	Surface sculpturing ornamentation:1-granulate/2-micro-granulate/3-psilate/ 4-verrucated.
*Abutilon grandifolium*	115.4	±20.6	116.1	±21.8	99.40	1	116.1	2	1	2	2	1	1	2	1
*Abutilon pannosum*	82.5	±4.1	82.7	±2.8	99.75	1	82.7	1	1	2	2	1	1	2	1
*Abutilon theophrasti*	83.9	±4.6	87.9	±5.8	95.53	1	87.9	1	1	2	1	1	1	2	1
*Alcea rosea*	116.9	±3.5	115.2	±4.7	101.49	2	116.9	2	4	1	2	2	2	5	2
*Malva multiflora*	178.8	±12.4	178.4	±5.6	100.85	3	178.8	2	4	1	1	2	2	5	2
*Malva neglecta*	79.04	±4.1	76.2	±3.6	103.72	2	79.04	1	4	1	1	2	2	5	3
*Malva nicaeensis*	132.2	±9.1	128.3	±8.4	103.03	2	132.2	2	4	1	1	2	2	5	3
*Malva parviflora*	77.6	±4.1	78.6	±5.3	98.75	1	78.6	1	4	1	1	2	2	5	2
*Malva sylvestris*	168.1	±11.5	163.8	±9.8	102.64	2	168.1	2	4	1	1	2	2	5	3
*Malvastrum coromandelianum*	152.7	±2.1	150.3	±2.2	101.62	2	152.7	2	2	2	2	1	1	4	1
*Sida alba*	76.3	±6.6	75.3	±6.4	101.35	2	76.3	1	1	1	2	1	1	4	1
*Gossypium barbadense*	103.5	±8.4	101.3	±8.7	102.19	2	103.5	2	3	1	2	1	1	2	1
*Abelomochus esculentus*	146.7	±10.2	142.3	±9.7	103.12	2	146.7	2	3	1	1	1	1	3	4
*Hibiscus sabdariffa*	168.8	±5.4	169.3	±6.08	99.7	1	169.3	2	3	1	1	1	2	5	1
*Hibiscus trionum*	185.1	±19.1	176.9	±19.7	104.57	2	185.1	2	3	1	1	1	2	5	1

### Start codon target scot analysis

**Fig. 11:**
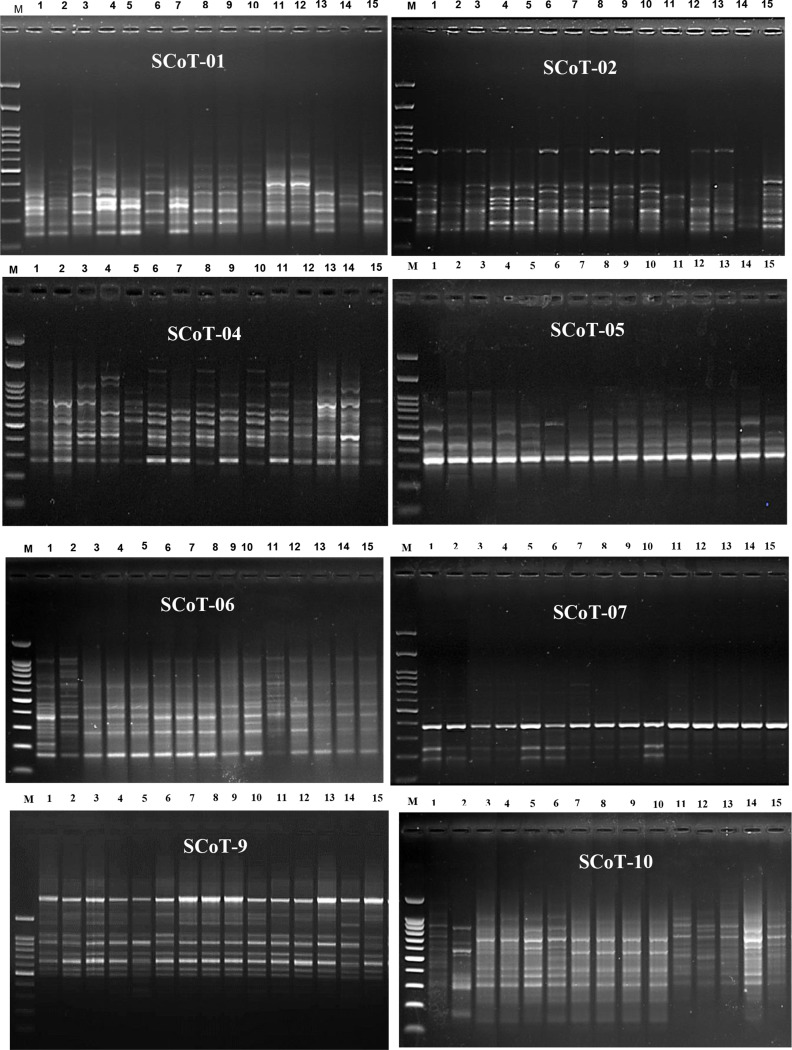
Band profiling of start codon targeted SCoT markers for 15 species of family Malvaceae

The amplification of specific sequence of 15 studied species of Malvaceae was examined using eight selected SCoT primers (Table 8 and Fig. 11). The total number of amplified fragments by all primers in the studied species was 100 fragments, including 51 polymorphic fragments with a ratio of 51%. The highest polymorphism among the studied species 72.73% was observed with the SCoT-06 primer, in which 8 polymorphic primers were detected. The lowest polymorphism 16.67% with the lowest number of amplicons only 6 amplified fragments was obtained in the SCoT-07 primer. Five of the eight primers showed polymorphism over 50%. In three SCoT primers SCoT-01, SCoT-06, and SCoT-10, no unique fragments were detected, while the other five primers yielded one unique band. The number of unique amplicons was the highest observed in *Lavatera cretica* of 2 fragments, which are emitted by primers SCoT-05 and SCoT-09. The positive unique band produced by the SCoT-02 primer was observed in *Hibiscus sabdariffa*; the unique fragment of primer SCoT-04 was obtained in *Alcea rosea*; whereas the unique amplicon of SCoT-07 was located in *Malva nicaeensis*.

There are four common bands in SCoT-10 primer for *Gossypium barbadense*, *Abelomoschus esculentus*, *Hibiscus trionum* and *H. sabdariffa*, these bands are; No. 2 (1175 bp), No. 6 (722 bp), No. 14 (217 bp), and No. 15 (196 bp). Band No. 1 in SCoT-05 primer with a molecular size of 1100 bp was specific for *Abutilon grandifolium*,* A. pannosum*,* A. theophrasti* and *Sida alba*.

**Table 8:** Amplification and polymorphism frequency resulted from scot analysis of some studied species of malvaceae

Primer code	Number of bands	Monomorphic bands	Polymorphic bands		Percentage of polymorphism
			Unique bands	Non unique bands	
SCoT − 01	13	4	-	9	69.23%
SCoT − 02	11	4	1	6	54.54%
SCoT − 06	11	3	-	8	72.73%
SCoT − 04	16	6	1	9	56.25%
SCoT − 05	10	3	1	6	60%
SCoT − 07	6	4	1	1	16.67%
SCoT − 9	16	11	1	4	25%
SCoT − 10	17	9	-	8	47.1%

### Numerical analysis

#### Macro and micromorphological characters

**Fig. 12:**
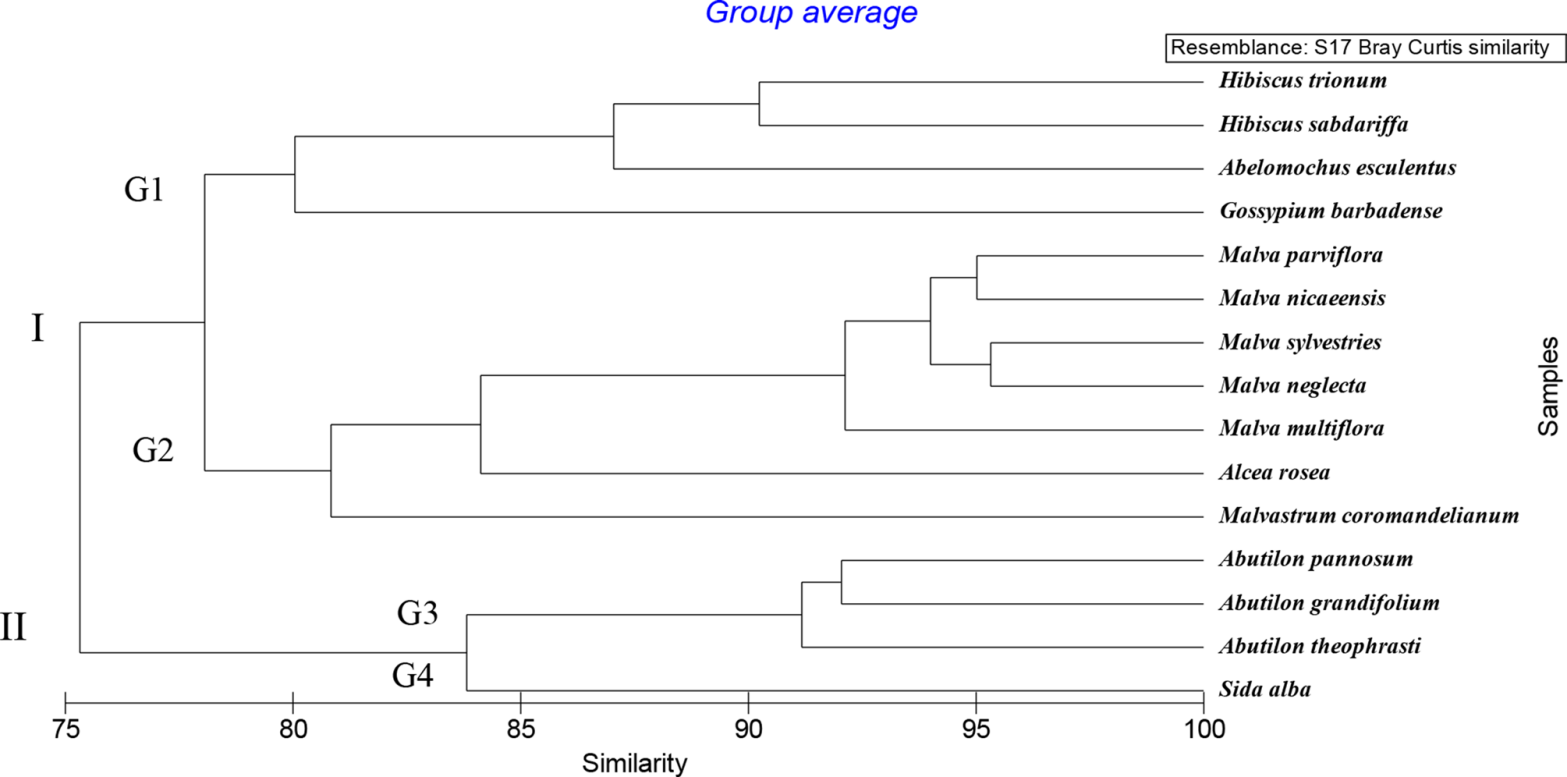
Dendrogram of the relationship between the 15 species of family Malvaceae based on macro and micro-morphological features, determined using PRIMER analysis

The morphological characters involved 66 character states for 15 species of Malvaceae. In the dendrogram produced from PRIMER software, version 6.0 analysis used similarity measure Bray Curtis and the group average (Fig. 12) showed that the 15 species were clustered into two main clusters at 75.32%.

The first cluster I consist of 11 species and divided into two groups at 78.07%. Group 1 involves 4 species, *Hibiscus trionum*,* H*. *sabdariffa*,* Abelomochus esculentus*, and *Gossypium barbadense*. Group 2 contains 7 species *Malva parviflora*, *M*. *nicaeensis*, *M*. *sylvestries*,* M*. *neglecta*,* M. multiflora*,* Malvastrum coromandelianum*, and *Alcea rosea*.

Where the second cluster II comprises 4 species and is divided into two groups at 83.82%. Group 3 consists of 3 species: *Abutilon theophrasti*,* A. grandifolium*, and *A. pannosum*, while group 4 contains one species: *Sida alba*.

#### Molecular characters

**Fig. 13:**
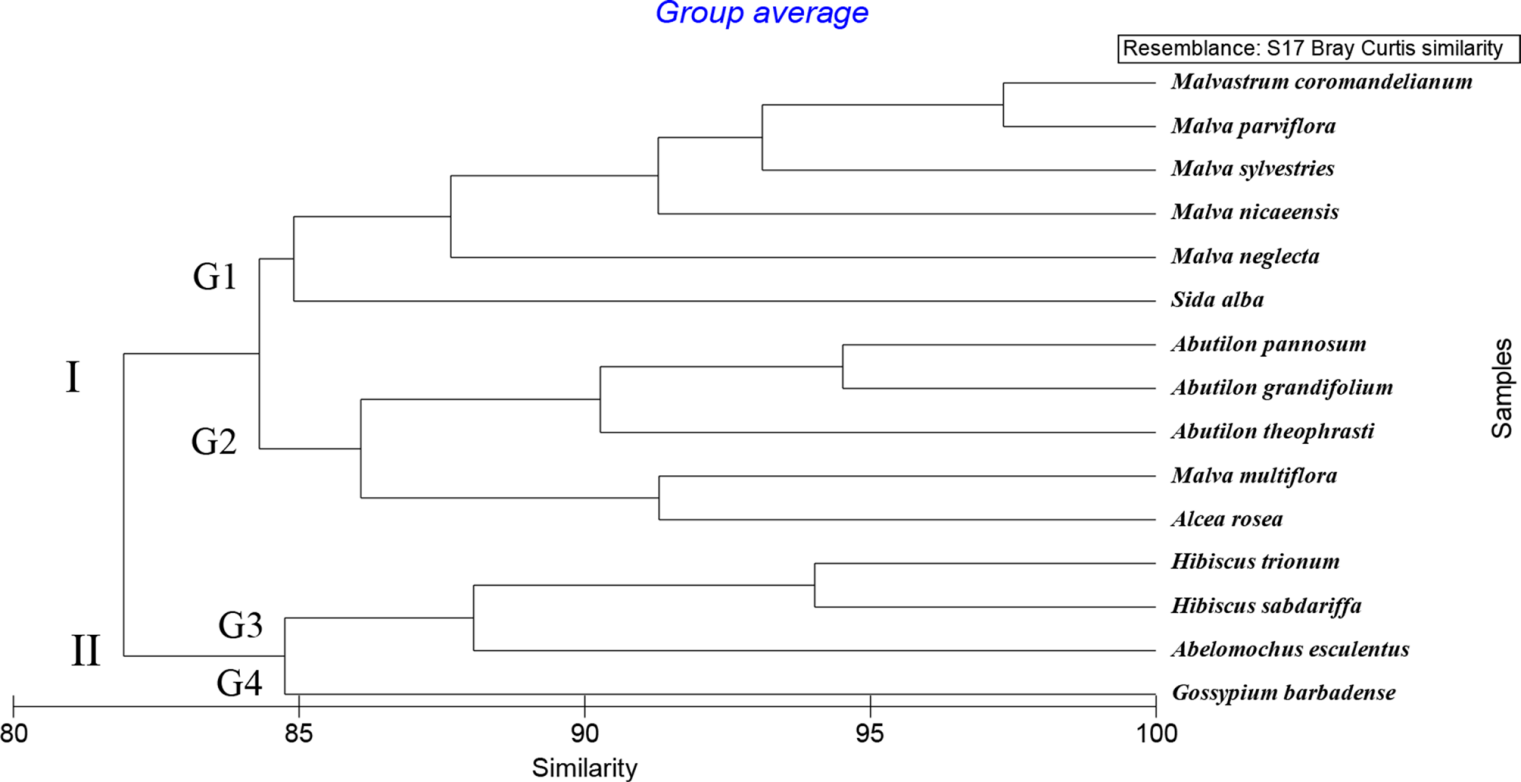
Dendrogram of the relationship between the 15 species of family Malvaceae based on Start Codon Targeted SCoT markers, determined using PRIMER analysis

The molecular characters involved 100 character states for 15 species of Malvaceae. The dendrogram produced from the PRIMER program, version 6.0 using similarity measure Bray Curtis and group average Fig. 13 showed that 15 species were grouped into two main clusters at 81.93%. The first cluster I include 11 species and divided into two groups at 84.31%. Group 1 comprises 6 species, *Malvastrum coromandelianum*, *Malva parviflora*, *M*. *sylvestries*,* M*. *nicaeensis*,* M*. *neglecta*, and *Sida alba*. Group 2 includes 5 species *Abutilon pannosum*, *A*. *grandifolium*, *A*. *theophrasti*, *Malva multiflora*, and *Alcea rosea.*

The second cluster II contains 4 species and is divided into two groups at 84.75%. Group 3 contains 3 species: *Hibiscus trionum*, *H*. *sabdariffa*, and *Abelomochus esculentus*, while group 4 has one species: *Gossypium barbadense*.

### Combined data morphological and molecular characters

A combination of macro and micro-morphological features and molecular characters was performed, 165-character states, which was used to occupy the cluster analysis to evaluate the relationships between the 15 studied species, using Hierarchical Cluster analysis using PRIMER software, version 6.0 analysis program used similarity measure Bray Curtis and group average.

**Fig. 14:**
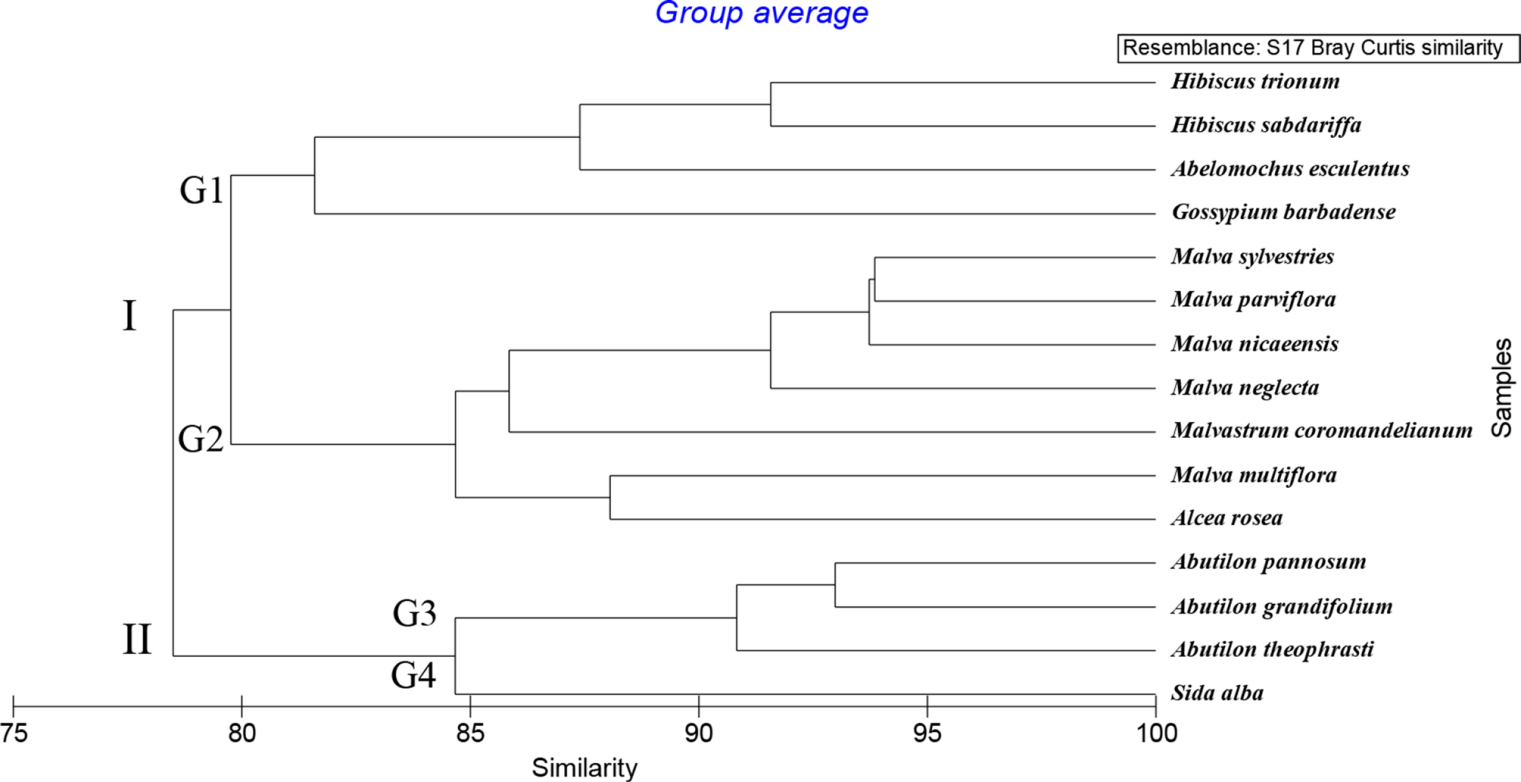
Dendrogram of the relationship between the 15 species of family Malvaceae based on combined macro and micro-morphological features and start codon targeted SCoT markers, determined using PRIMER analysis

The dendrogram in (Fig. 14) showed that the 15 species were gathered into two major clusters at 78.5%. The first cluster I consists of 11 species, which are divided into two groups at 79.77%. Group 1 comprises 4 species: *Hibiscus trionum*,* H*. *sabdariffa*,* Abelomochus esculentus*, and *Gossypium barbadense*. Group 2 comprises 7 species, *Malva parviflora*,* M*. *nicaeensis*, *M*. *sylvestries*, *M*. *neglecta*, *M. multiflora*, *Malvastrum coromandelianum*, and *Alcea rosea*.

The second cluster I include 4 species and is divided into two groups at 84.67%. Group 3 consists of 3 species *Abutilon theophrasti*,* A*. *grandifolium*, and *A*. *pannosum*, while group 4 has one species: *Sida alba*.

## Discussion

These studies were based on habit, leaf morphology, calyx, carpel morphology, pollen grain shape, size, ornamentation, and SCoT analysis. Given that environmental factors can exert a profound influence on plant morphology [65], the observed variation in characters such as leaf shape, trichome density, and stipule dimensions may partially reflect ecological plasticity rather than solely genetic divergence. Similarly, genetic structure can significantly influence various agronomic and phenotypic traits in plants [66, 67]. One of the most important characteristics employed for determining the phylogenetic interpretation within the tribes and species of the Malvaceae family is the presence or absence of hypocalyx [11].

Pollen grain characters presented useful information for evaluating the taxonomy of Malvaceae [34]. Pollen grain shapes in studied species are extended between three shapes; oblate-spheroidal, prolate-spheroidal, and spheroidal, the pollen size large (50–99 μm) located in five species and very large (100–199 μm) in twelve species, these results are matching with the findings of different studies for some species of Malvaceae [68, 33, 69, 70, 71, 34].

In the present study, the dendrogram of combined data macro and micro-morphological features and start codon targeted SCoT markers revealed the division of the studied species into two clusters. From the results of the cluster analysis of combined data, we recommend separation between species belong to tribe Sideae (*Abutilon theophrasti*,* A. pannosum*,* A. grandifolium* and *Sida alba*) from species belong to tribe Malveae (*Malva parviflora*,* M. sylvestris*,* M. nicaeensis*,* M. neglecta*,* M. aegyptia*,* M. multiflora*,* Malvastrum coromandelianum* and *Alcea rosea*), these species in the tribe Abutileae are differed from Malveae species in some phenotypic and molecular characteristics such as; absence of hypocalyx, yellow or off-white petals, spines in pollen grains are short, and presence of a specific DNA band number one at the primer SCoT-05 with a molecular size of 1100 bp these result supports and agrees with Reveal [72] who separated the subtribes Abutilinae and Sidinae from Malveae under the tribe Sidieae this result is consistent with Figs. 12 and 14, which show that cluser II consists of two genera, *Abutilon* and *Sida*. Similarly, Tate et al. [73] studied the phylogenetic relations within the Malveae tribe based on sequence data from ITS regions and accepted two main clades: one comprising of *Abutilon* and *Sida* alliance and a second covering the rest of species.

On the other hand, species of the tribe Hibisceae, *Abelmoschus esculentus*,* Hibiscus sabdariffa* and *H. trionum* are more related to each other and characterized by; stem nature ererct, leaf composition lobed, leaf venation palmately, flower solatory, hypocalyx present, anther crowded at tip, ovary shape ovate, and pollen size very large, while the tribe Gossypieae differ in; stipule shape lanceolate, leaf texture glaberscent, leaf apex acuminate, pedicel surface glabrous, apex of hypocalyx toothed, number of hypocalyx three, ovary surface glabrous, stigma type slightly clavate, and stigma number three. These results also provide support for the monophyletic of the Malvaceae. The relation supports the view of Reveal [72] that placed them in two separate tribes, Gossypieae and Hibisceae, respectively.

The present results also showed that the dendrogram of molecular analysis revealed the division of the studied species into two clusters. The first cluster I includes 11 species: *Malvastrum coromandelianum*, *Malva parviflora*, *M*. *sylvestries*,* M*. *nicaeensis*,* M*. *neglecta*, *Sida alba*, *Abutilon pannosum*, *A*. *grandifolium*, *A*. *theophrasti*, *Malva multiflora*, and *Alcea rosea* that representing tribe Malvae. The second cluster II contains 4 species and is divided into two groups. Group one includes 3 species: *Hibiscus trionum*, *H*. *sabdariffa*, and *Abelomochus esculentus* that representing tribe Hibisceae, while group two has one species *Gossypium barbadense* tribe Gossypieae, this agrees with Takhtajan [74], APG IV [75], Kubitzki and Bayer [76], and Colli-Silva et al. [77]. Additionally, this result of the dendrogram from the Start codon targeted (SCoT) analysis supports and agrees with the previous study by Elkhiat et al. [78].

Based on numerical analysis, this study provided nearly sufficient information on the taxonomic importance of macro- and micro-morphological characters and Start codon targeted SCoT analysis, confirming its efficiency in separating and distinguishing the studied species. Finally, the present result agrees and support the view of the classification of Reveal [72] and APGIV [75].

## Conclusion

The results showed that macro- and micro-morphological characteristics and molecular analysis, notably the morphological characteristics, are important for recognizing and differentiating the species under consideration here. The selected species of Malvaceae differences and affinities have been fairly described by the morphological characterization used in this study. Also, it is crucial to distinguish between species based on the habit, stem, types of leaf, inflorescence, and pollen grain. The outcome of the numerical analysis demonstrates that morphological traits are crucial in discriminating between species that belong to the same genus. Additionally, the morphological character dendrogram and the phylogenetic analysis of the SCoT indicated general concordance. Integrating traditional botanical methods with advanced imaging technologies provides a strong foundation for future research focused on the sustainable utilization and conservation of these ecologically and medically significant species. The results indicated that the characteristics of macro- and micro-morphological characters and Start codon targeted Scot analysis would be helpful for the identification and differentiation among the studied species. The traits assessed in this study were consistent with previous classifications of the Malvaceae Reveal [72] and APG IV [75].

## Electronic supplementary material

Below is the link to the electronic supplementary material.

Supplementary Material 1

Supplementary Material 2

Supplementary Material 3

Supplementary Material 4

## Data Availability

The datasets produced and/or analyzed during this study are available from the corresponding author upon reasonable request.
