# Supplementary material for: Taxonomic significance of morphological and molecular variation in Egyptian Malvaceae species
Source: BMC Plant Biol. 2025 May 16;25:646. doi: 10.1186/s12870-025-06609-4 (PMC12082894; doi:10.1186/s12870-025-06609-4)
Supplement: Supplementary file 1 — Supplementary Material 1 [file 12870_2025_6609_MOESM1_ESM.docx]

| **Character list for the numerical analysis of the studied species of malvaceae.** | |
| --- | --- |
| **Growth form** | 1. Duration: annual [1]/perennial [2]. |
|  | 1. Habit: herb [1] /shrub [2]. |
| **Stem** | 1. Nature: erect [1] /erect to decumbent [2]. |
|  | 1. Branching: branched [1] /unbranched [2]. |
|  | 1. Texture: pubescent [1]/ tomentose [2] /glabrescent [3]. |
|  | 1. Color: green [1] / red [2]. |
|  | 1. Surface: smooth [1] / rough [2]. |
|  | 1. Internode length (cm): very short (1.7-2.5 cm) [1]/ short (3-4.5 cm) [2]/long (5-7 cm) [3] /very long (more than 7 cm) [4]. |
| **Stipule** | 1. Stipule: normal [1] /membranous [2]. |
|  | 1. Shape: lanceolate [1] /triangular [2] /ovate [3] /linear [4]. |
|  | 1. Apex: acute [1] /acuminate [2]. |
|  | 1. Texture: pubescent [1] / tomentose [2] / glabrous [3]. |
|  | 1. Dark veins: present [1] / absent [2]. |
|  | 1. Length (mm): short (3.2-7 mm) [1] / long (9-12 mm) [2] /very long (more than 12 mm) [3]. |
|  | 1. Width (mm): narrow (0.3-2.3 mm) [1] / wide (3.5-5.2 mm) [2]. |
| **Leaf** | 1. Petiole surface: pubescent [1] / tomentose [2] / glabrous [3]. |
|  | 1. Petiole length(cm): short (1.4-4.5 cm) [1] / long (6-8.6 cm) [2] /very long (10-14.2 cm) [3]. |
|  | 1. Blade composition: entire [1] / lobed [2]. |
|  | 1. Blade shape: entire [1] /palmatifid [2]/palmatisect [3]/palmatipartite [4] /Pinnatifid [5] |
|  | 1. Blade segment: lobed [1] /entire [2] /absent [3]. |
|  | 1. Blade venation: pinnately nerved [1] / Palmately nerved [2]. |
|  | 1. Blade apex: acute [1] / acuminate [2] /obtuse [3]. |
|  | 1. Blade margin: dentate [1] / lobate [2] /serrate [3] / crenate [4] /entire [5]. |
|  | 1. Blade texture: pubescent [1] / tomentose [2] /glabrescent [3]. |
|  | 1. Blade length (cm): short (1.3-4.5 cm) [1] /long (5-6.5 cm) [2] /very long (8-13.4 cm) [3]. |
|  | 1. Blade width (cm): (1.7-3.2 cm) [1] / (4-7 cm) [2] / (more than 7 cm) [3]. |
| **Inflorescence** | 1. Flower composition: solitary [1] /racemose [2] /cyme monochasial [3]. |
| **Flower** | 1. Pedicel surface: pubescent [1] /tomentose [2] /glabrous [3]. |
|  | 1. Pedicel length (cm): very short (0.2-0.9 cm) [1] /short (1-1.7 cm) [2] /long (2.3-4 cm) [3]. |
| **Hypocalyx** | 1. Hypocalyx: present [1] /absent [2]. |
|  | 1. Shape: linear [1]/oblong [2] /broad ovate [3] / lanceolate [4] /absent [5]. |
|  | 1. Apex: acute [1] /toothed [2] /absent [3]. |
|  | 1. Surface: pubescent [1] /glabrous [2] /absent [3]. |
|  | 1. Number: two [1] /three [2] /seven to ten [3] more than10[4] /absent [5]. |
| **Calyx** | 1. Color: green [1] /yellowish green [2]/ red [3]. |
|  | 1. Apex: acute [1] /acuminate [2] /obtuse [3]. |
|  | 1. Surface: pubescent [1] /tomentose [2] /glabrous [3]. |
|  | 1. Tube length (mm): short (0.1-0.4 mm) [1] / long (0.5-0.7 mm) [2] / very long (more than 0.7 mm) [3]. |
|  | 1. Sepal length (mm): very short (4.6-6.6 mm) [1]   /short (7-12.3 mm) [2] /long (more than 12.3 mm) [3]. |
| **Corolla** | 1. Petal color: yellow [1 /pale violet [2]/off white [3]/pink [4]/red [5]. |
|  | 1. Color: monomorphic [1]/dimorphic [2]. |
|  | 1. Purple spot: present /absent [2]. |
|  | 1. Petal apex: emarginate [1] /not emarginate [2]. |
|  | 1. Dark veins: present [1] /absent [2]. |
|  | 1. Claw: clawed [1] /not clawed [2]. |
| **Staminal tube** | 1. Surface: hairy [1] /glabrous [2]. |
|  | 1. Color: yellow [1] /purple to yellow [2] / pale yellow [3] /purple [4] /pink [5]. |
|  | 1. Anther: crowded at tip [1] /spread [2]. |
|  | 1. Length (cm): very short (0.4-0.8 cm) [1] /short (1-1.2 cm) [2] /long (1.6-3 cm) [3] |
| **Gynoecium** | 1. Ovary surface: tomentose [1] / pubescent [2] / glabrous [3]. |
|  | 1. Ovary shape: discoid [1] / sub globose [2] /ovate [3]. |
|  | 1. Ovary length (mm): very short (0.7-1.5 mm) [1] /short (2.5-3 mm) [2] / long (5-10 mm) [3]. |
|  | 1. Ovary width (mm): (1.4-2.5 mm) [1]/ (3-4 mm) [2] / (5-8 mm) [3]. |
|  | 1. Style length (mm): very short (0.8-2 mm) [1] /short (2.3-4.8 mm) [2] /long (6-10 mm) [3] /very long (more than 10 mm) [4]. |
|  | 1. Stigma type: terete [1] /discoid [2] /slightly clavate [3]. |
|  | 1. Stigma number: three [1] /five [2] / (8-10) [3] /more than 10 [4]. |
| **Pollen grain** | 1. Pollen shape: oblate- spheroidal (88-99) [1] / prolate-spheroidal (101-114) [2] / spheroidal (100) [3]. |
|  | 1. Pollen size (µm): large (50-99 µm) [1] /very large (100-199 µm) [2]. |
|  | 1. Spine length (µm): short (3.5-4.5 µm) [1] /long (5-8.8 µm) [2] /very long (more than 8.8 µm) [3]/variable (3.5-4.5 + 5-8.8 µm). |
|  | 1. Spine shape: conical [1] /flask shape [2]. |
|  | 1. Spine density: spread [1] /condensed [2]. |
|  | 1. Spine type: monomorphic [1] /dimorphic [2]. |
|  | 1. Spine base: bulbous [1] /flat [2]. |
|  | 1. Bulbous surface: smooth [1] / granulate [2] / spiny [3] /verrucated [4] / absent [5]. |
|  | 1. Surface sculpturing (ornamentation): granulate [1]/ micro-granulate [2] /psilate [3] /verrucated [4]. |
