# Supplementary material for: Taxonomic significance of morphological and molecular variation in Egyptian Malvaceae species
Source: BMC Plant Biol. 2025 May 16;25:646. doi: 10.1186/s12870-025-06609-4 (PMC12082894; doi:10.1186/s12870-025-06609-4)
Supplement: Supplementary file 2 — Supplementary Material 2 [file 12870_2025_6609_MOESM2_ESM.docx]

| **Primer Name** | **Sequence** |
| --- | --- |
| **SCoT-01** | 5'-ACGACATGGCGACCACGC-3' |
| **SCoT-02** | 5'-ACCATGGCTACCACCGGC-3' |
| **SCoT-04** | 5'-ACCATGGCTACCACCGCA-3' |
| **SCoT-05** | 5'-CAATGGCTACCACTAGCG-3' |
| **SCoT-06** | 5'-CAATGGCTACCACTACAG-3' |
| **SCoT-07** | 5'-ACAATGGCTACCACTGAC-3' |
| **SCoT-09** | 5'-ACAATGGCTACCACCAGC-3' |
| **SCoT-10** | 5'-ACAATGGCTACCACTACC-3' |
