# Supplementary material for: Taxonomic significance of morphological and molecular variation in Egyptian Malvaceae species
Source: BMC Plant Biol. 2025 May 16;25:646. doi: 10.1186/s12870-025-06609-4 (PMC12082894; doi:10.1186/s12870-025-06609-4)
Supplement: Supplementary file 4 — Supplementary Material 4 [file 12870_2025_6609_MOESM4_ESM.pptx]

## Slide 1
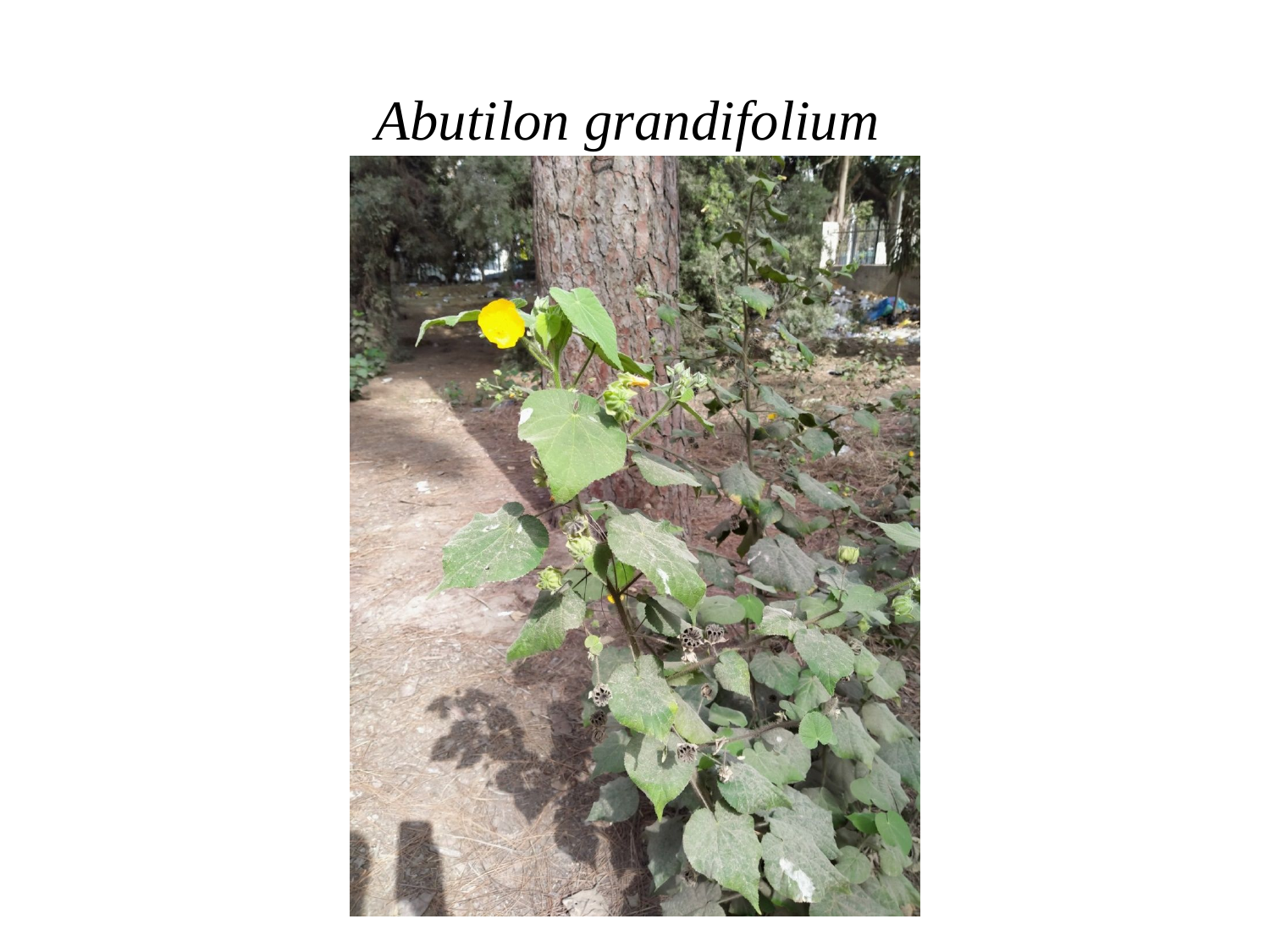

# Abutilon grandifolium

## Slide 2
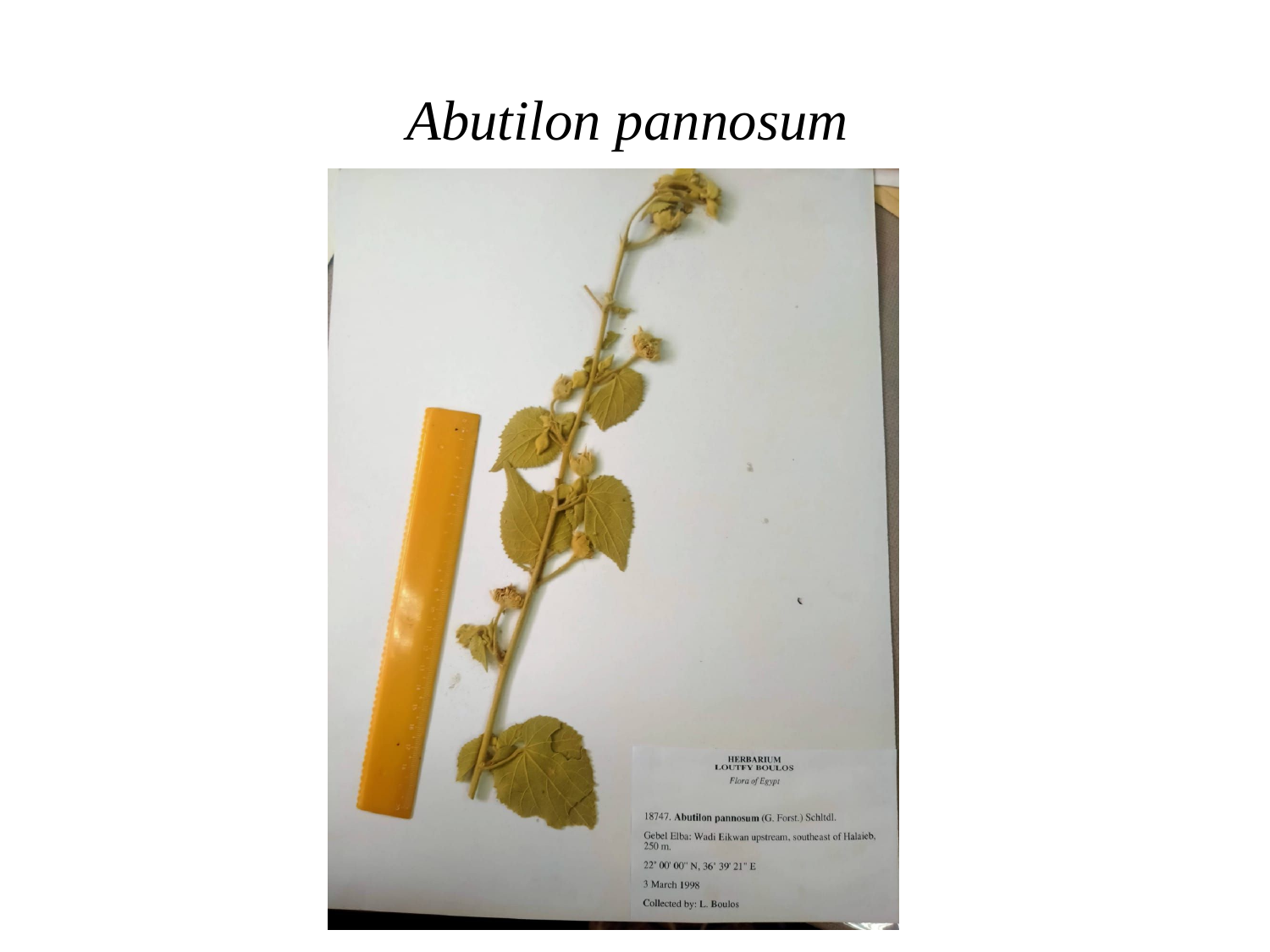

# Abutilon pannosum

## Slide 3
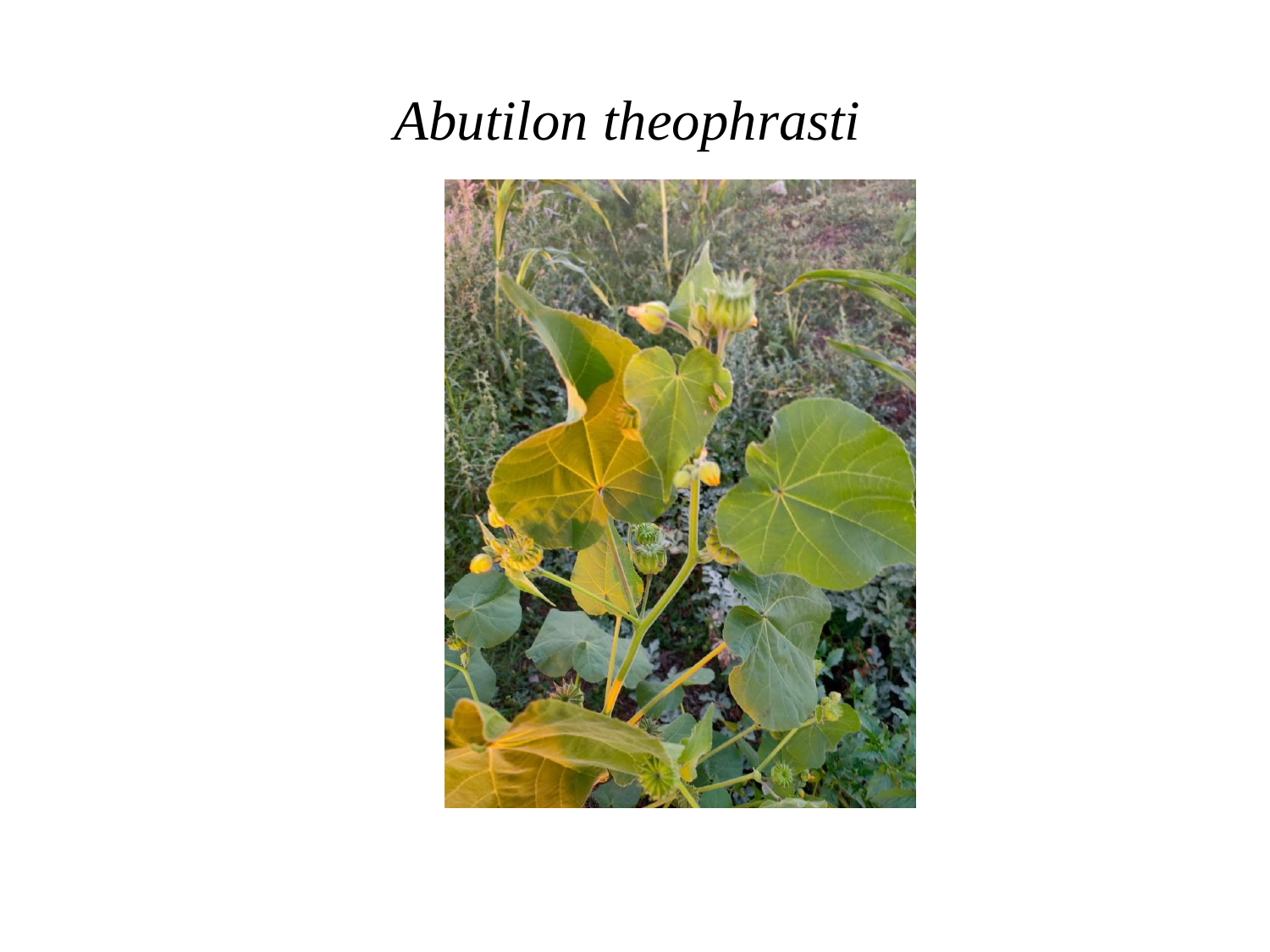

# Abutilon theophrasti

## Slide 4
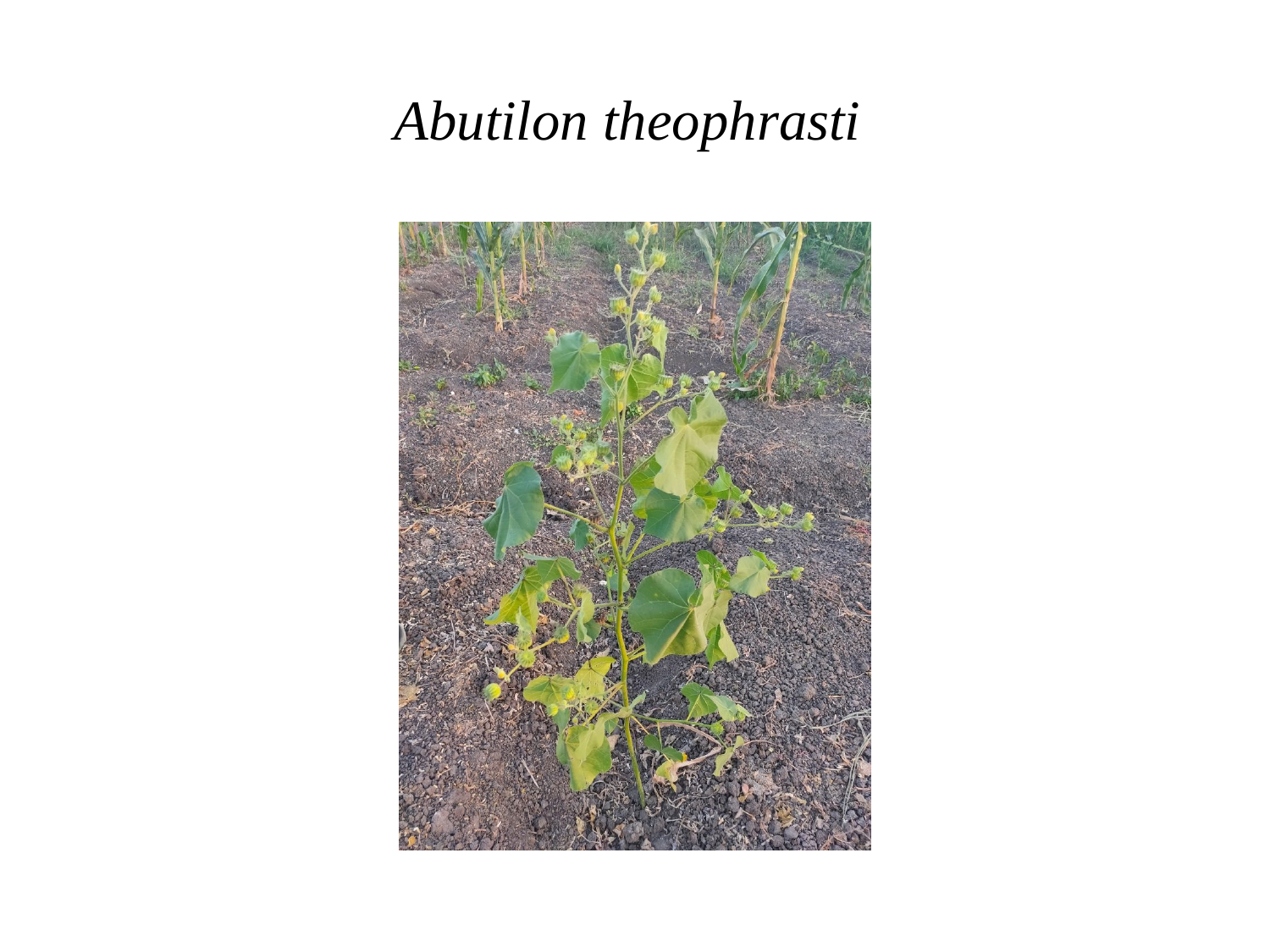

# Abutilon theophrasti

## Slide 5
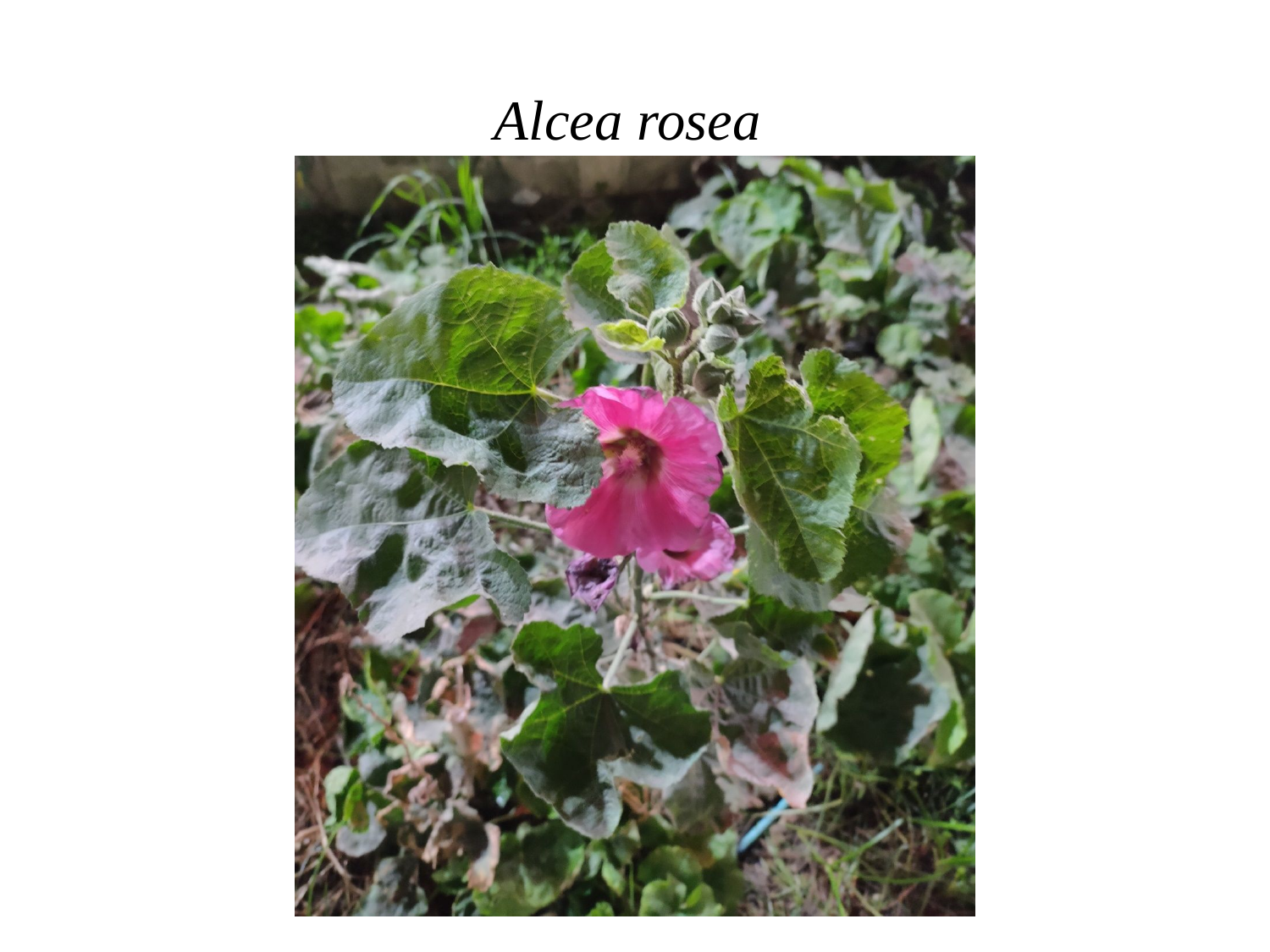

# Alcea rosea

## Slide 6
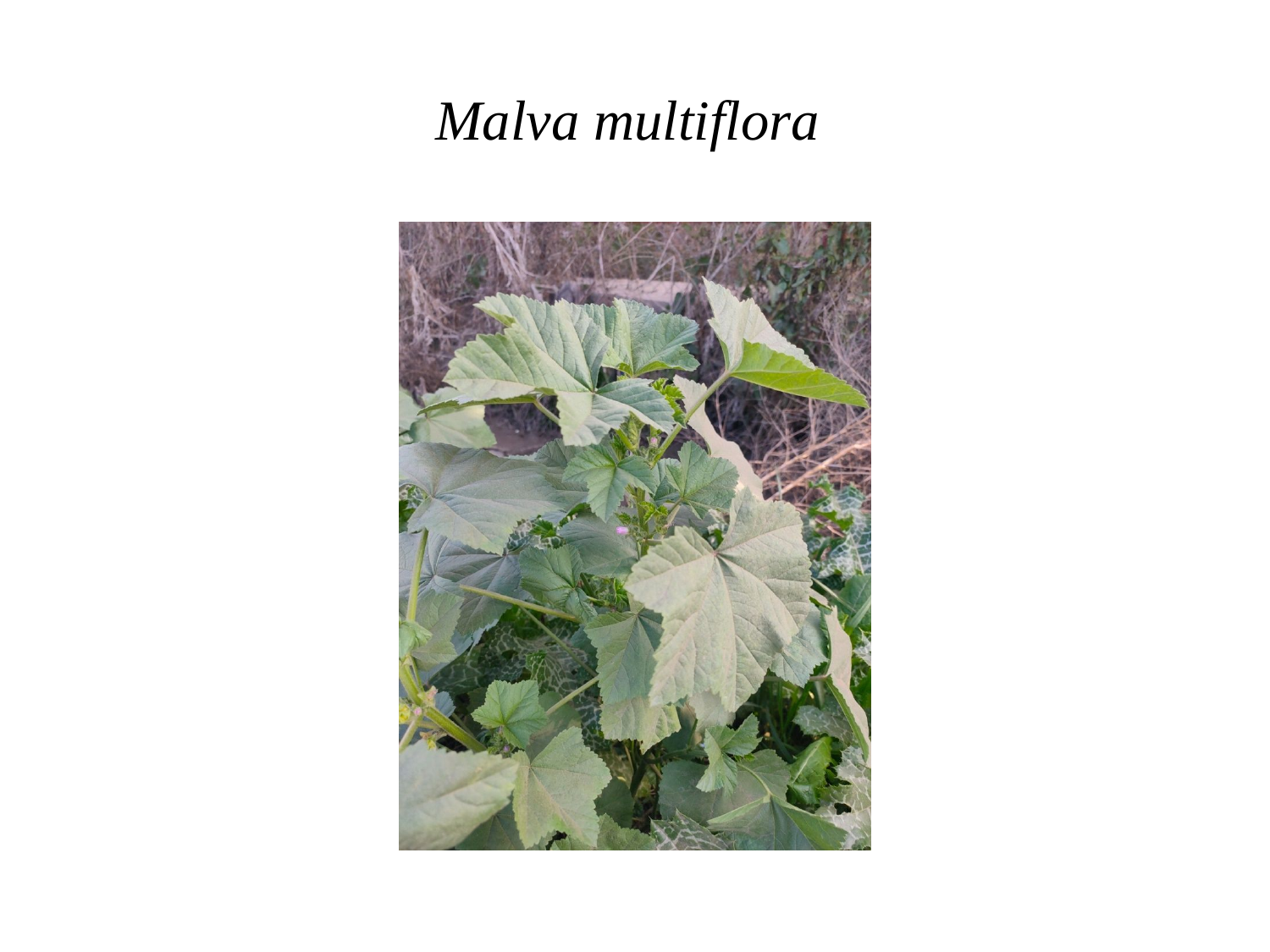

# Malva multiflora

## Slide 7
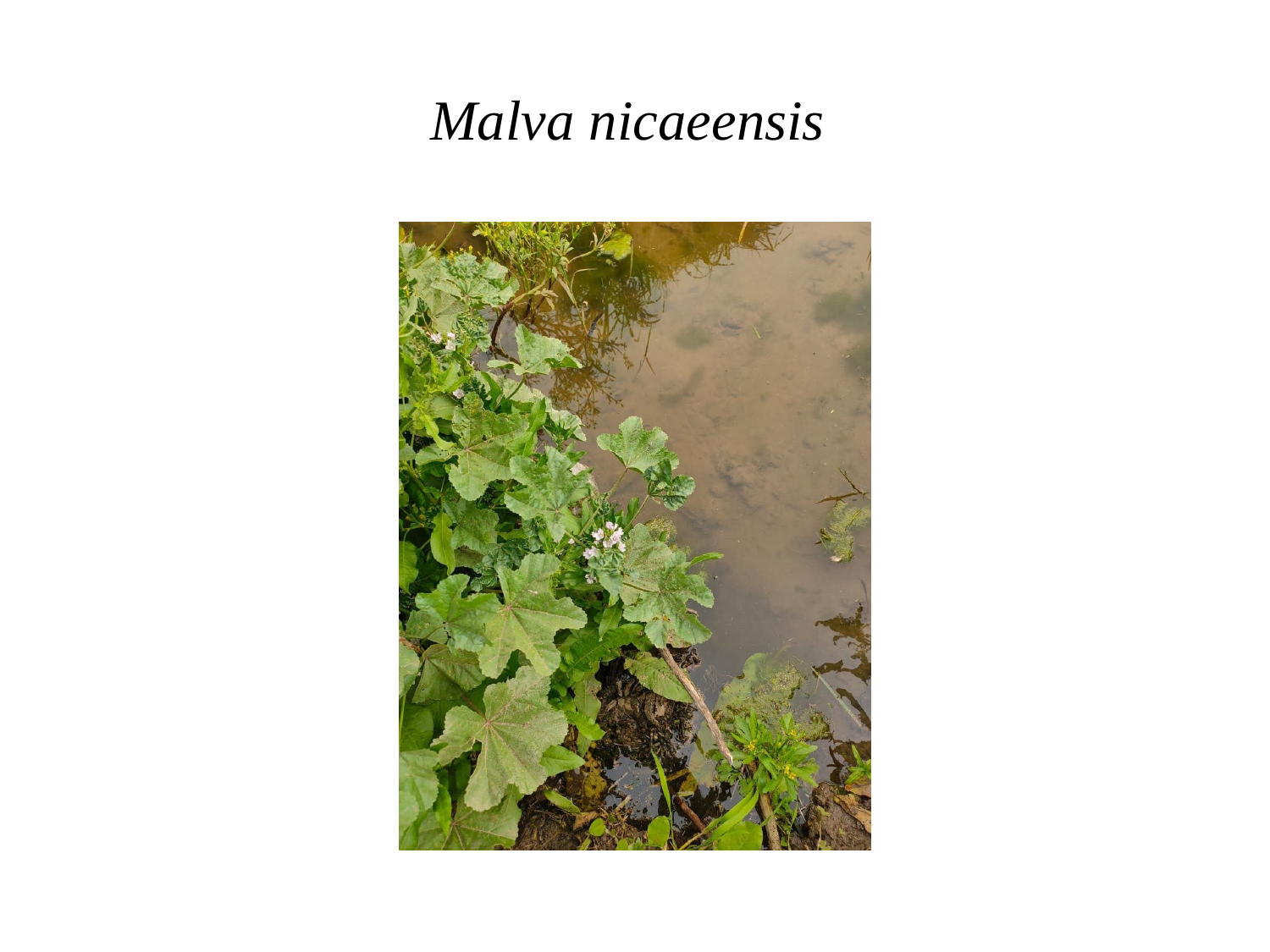

# Malva nicaeensis

## Slide 8
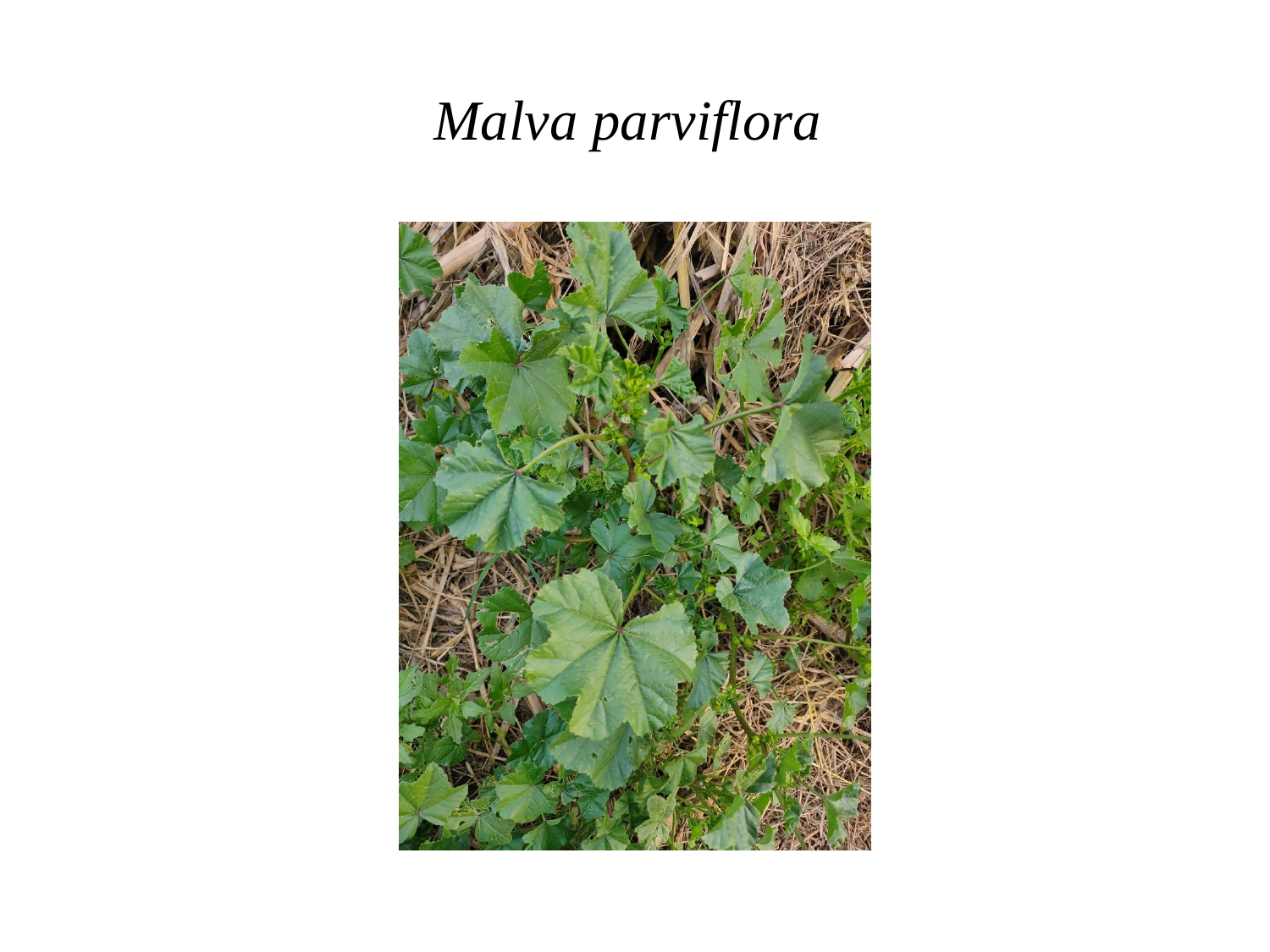

# Malva parviflora

## Slide 9
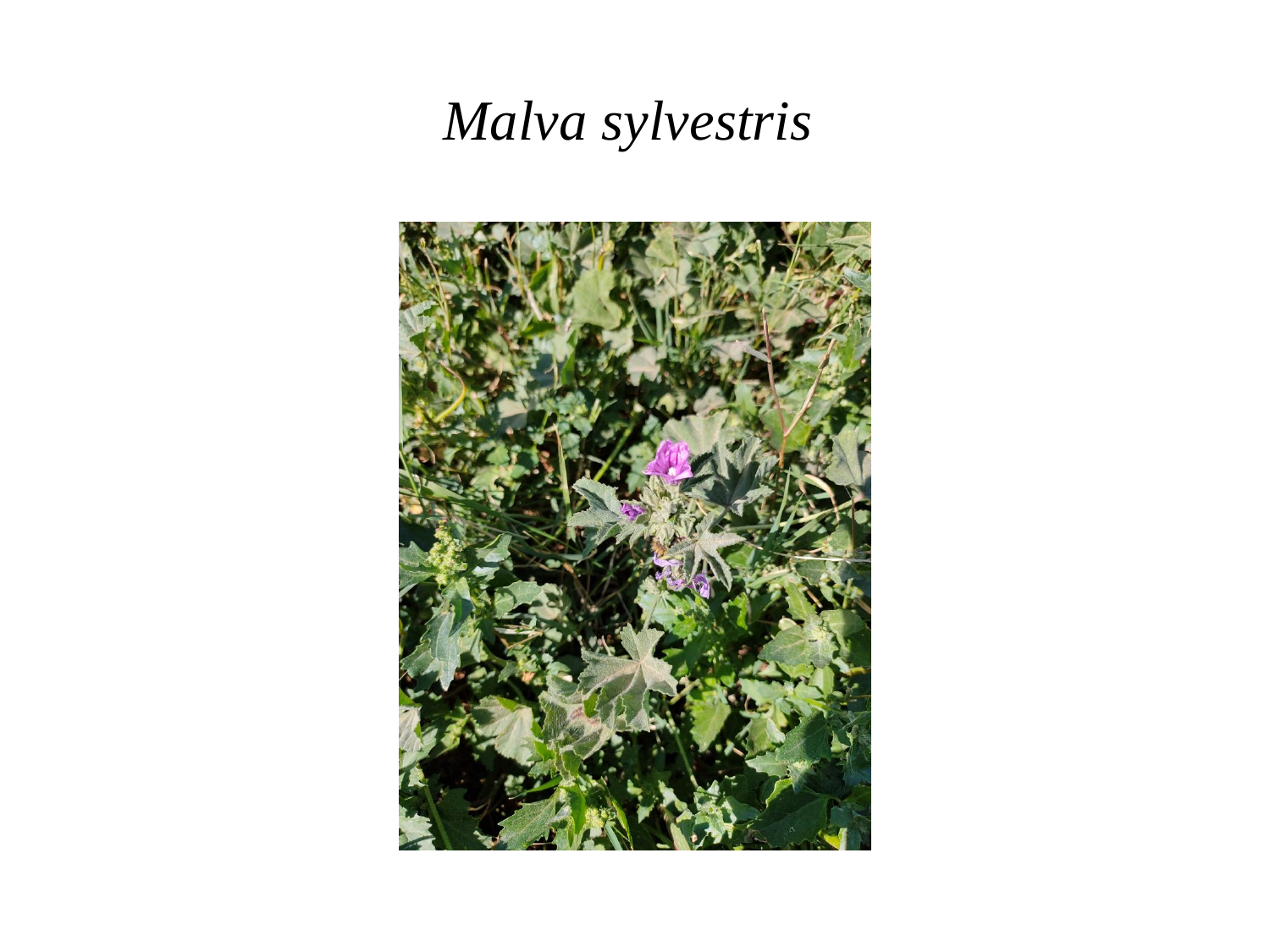

# Malva sylvestris

## Slide 10
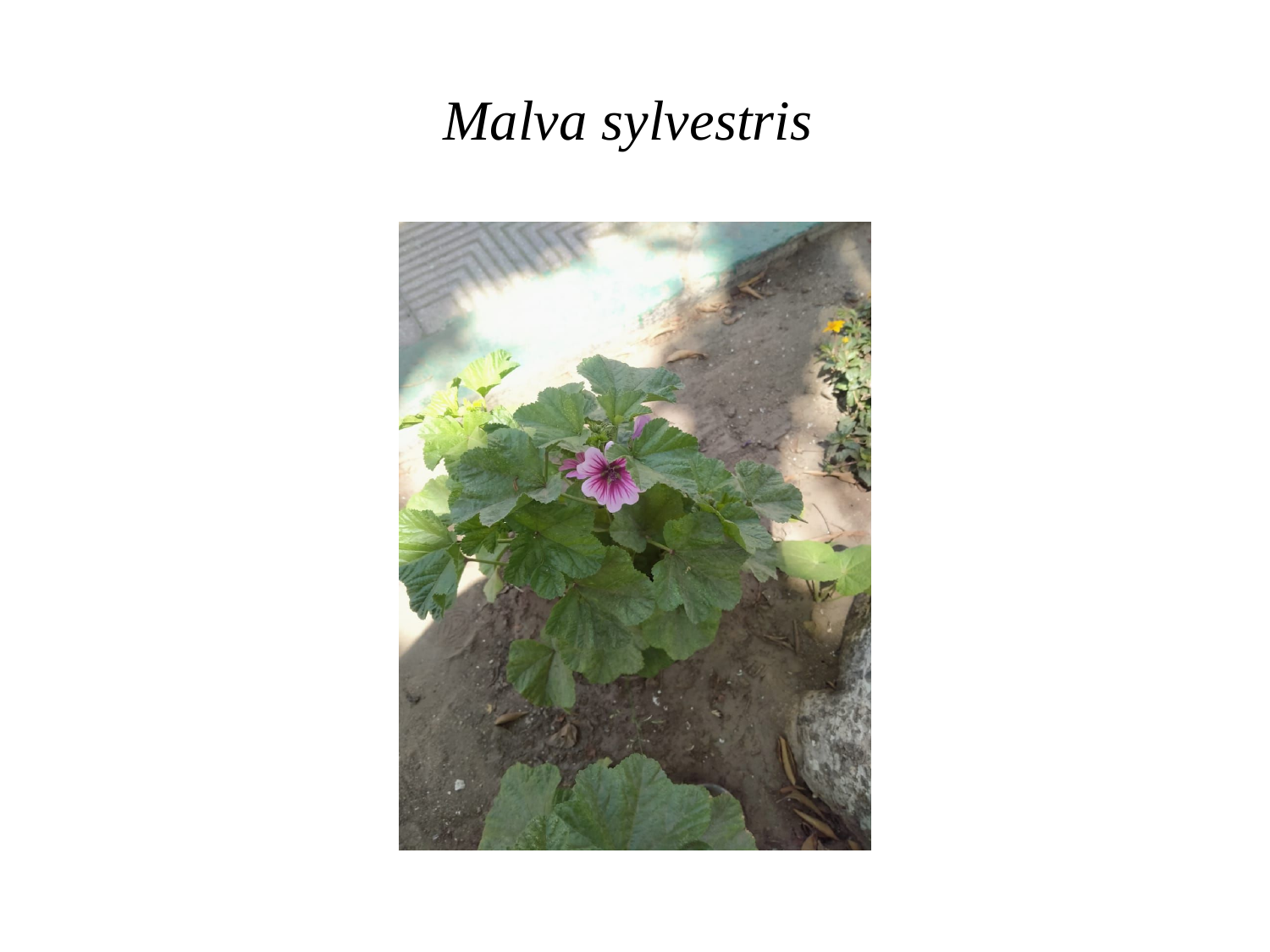

# Malva sylvestris

## Slide 11
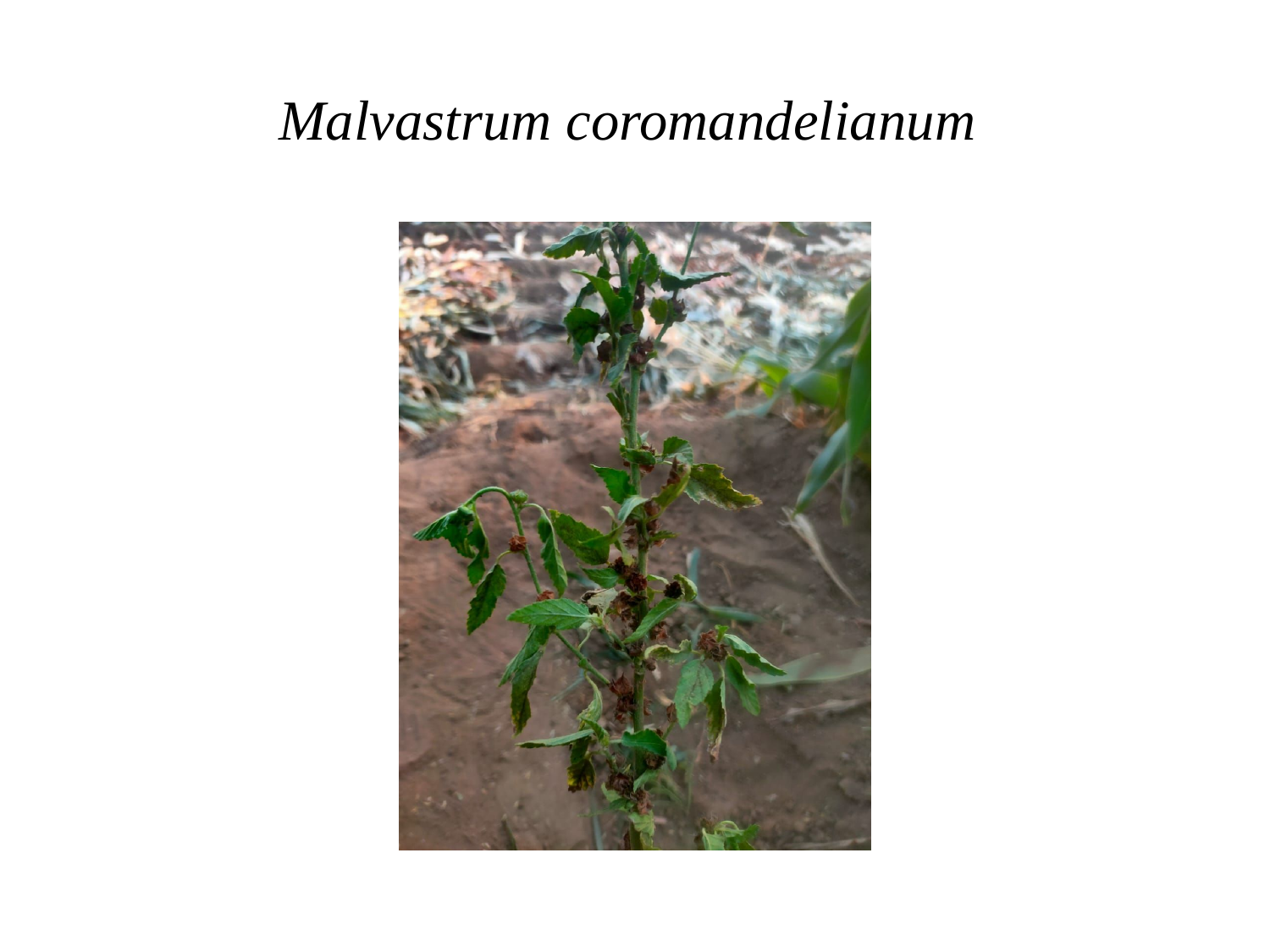

# Malvastrum coromandelianum

## Slide 12
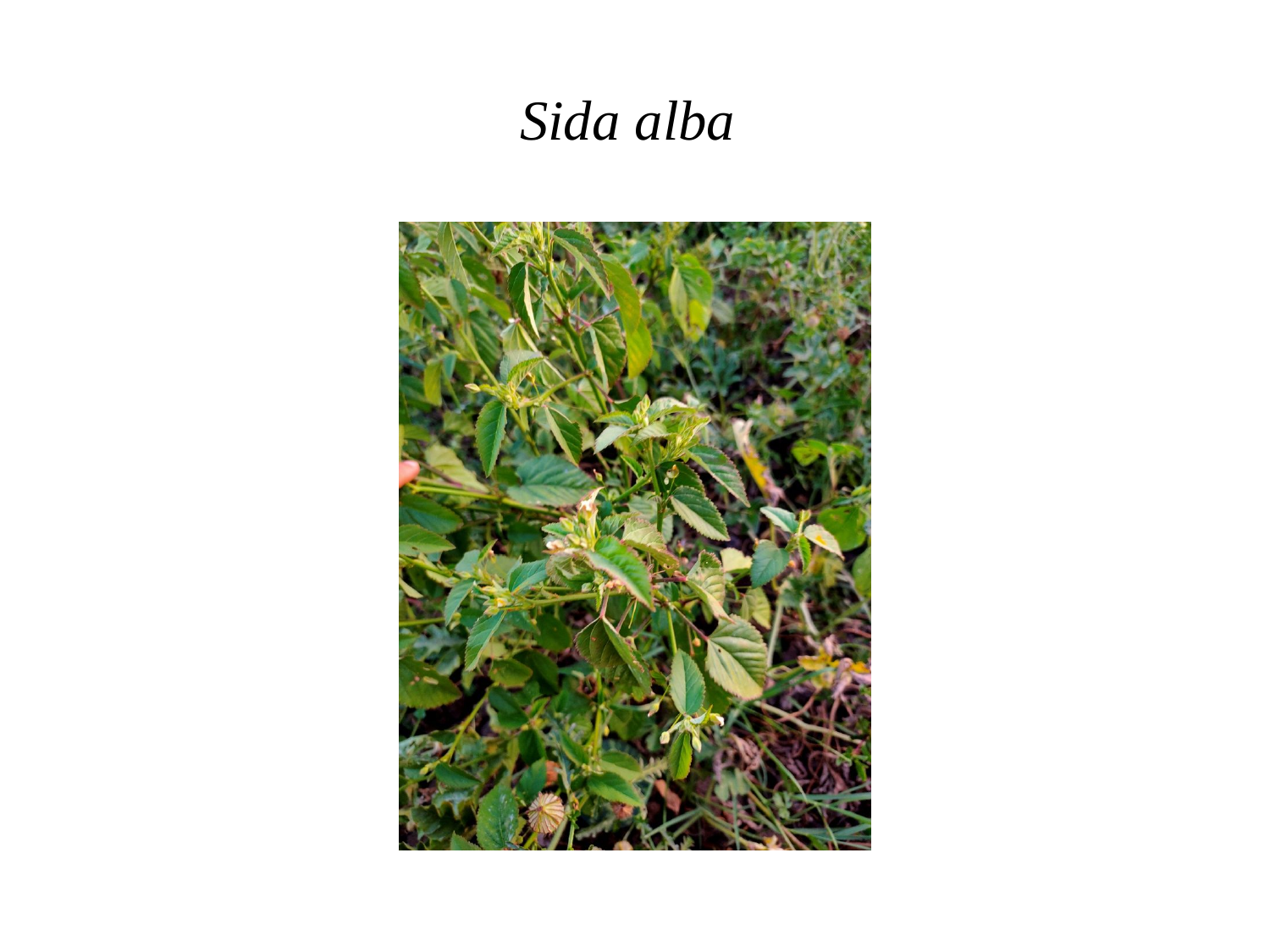

# Sida alba

## Slide 13
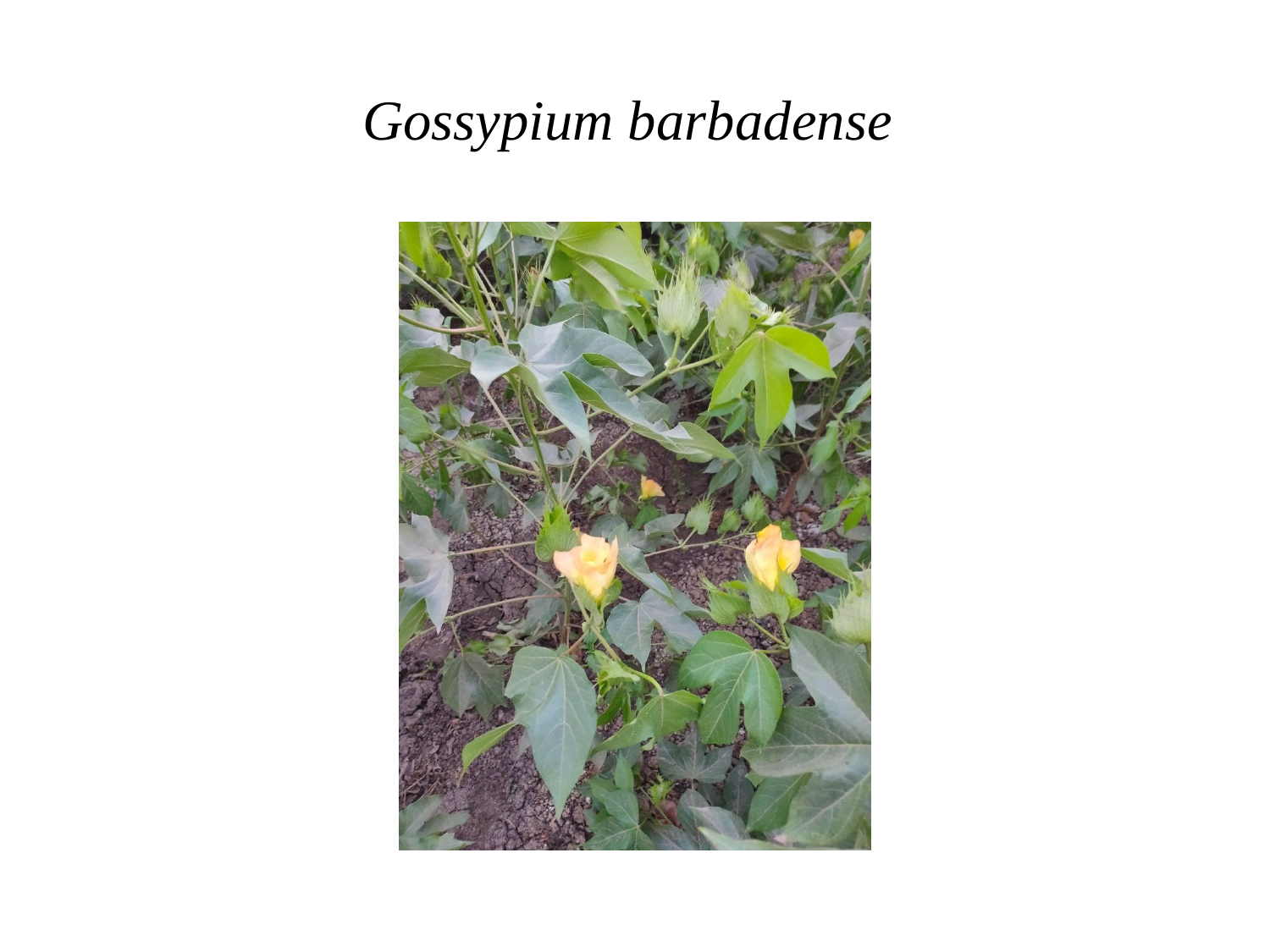

# Gossypium barbadense

## Slide 14
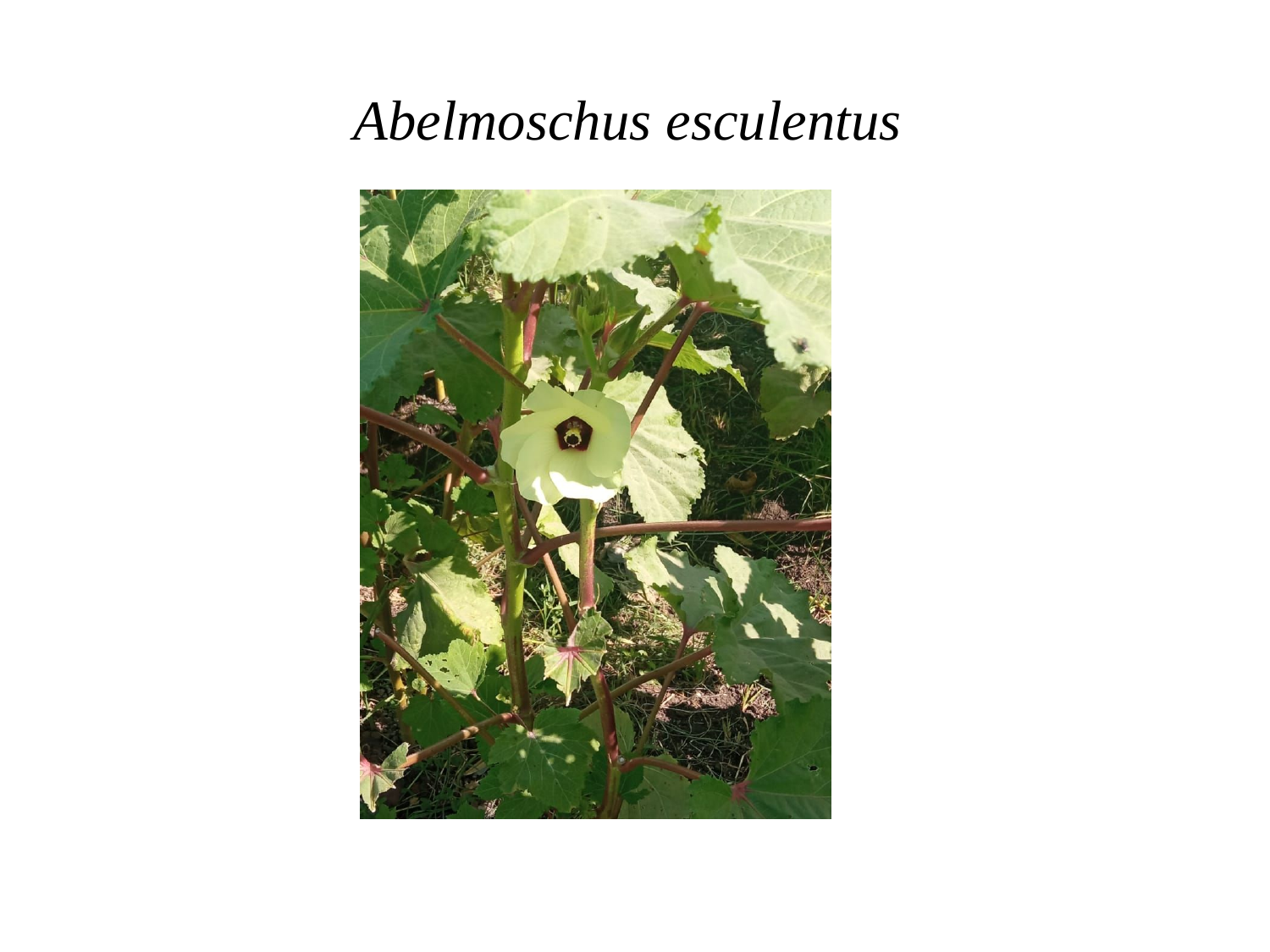

# Abelmoschus esculentus

## Slide 15
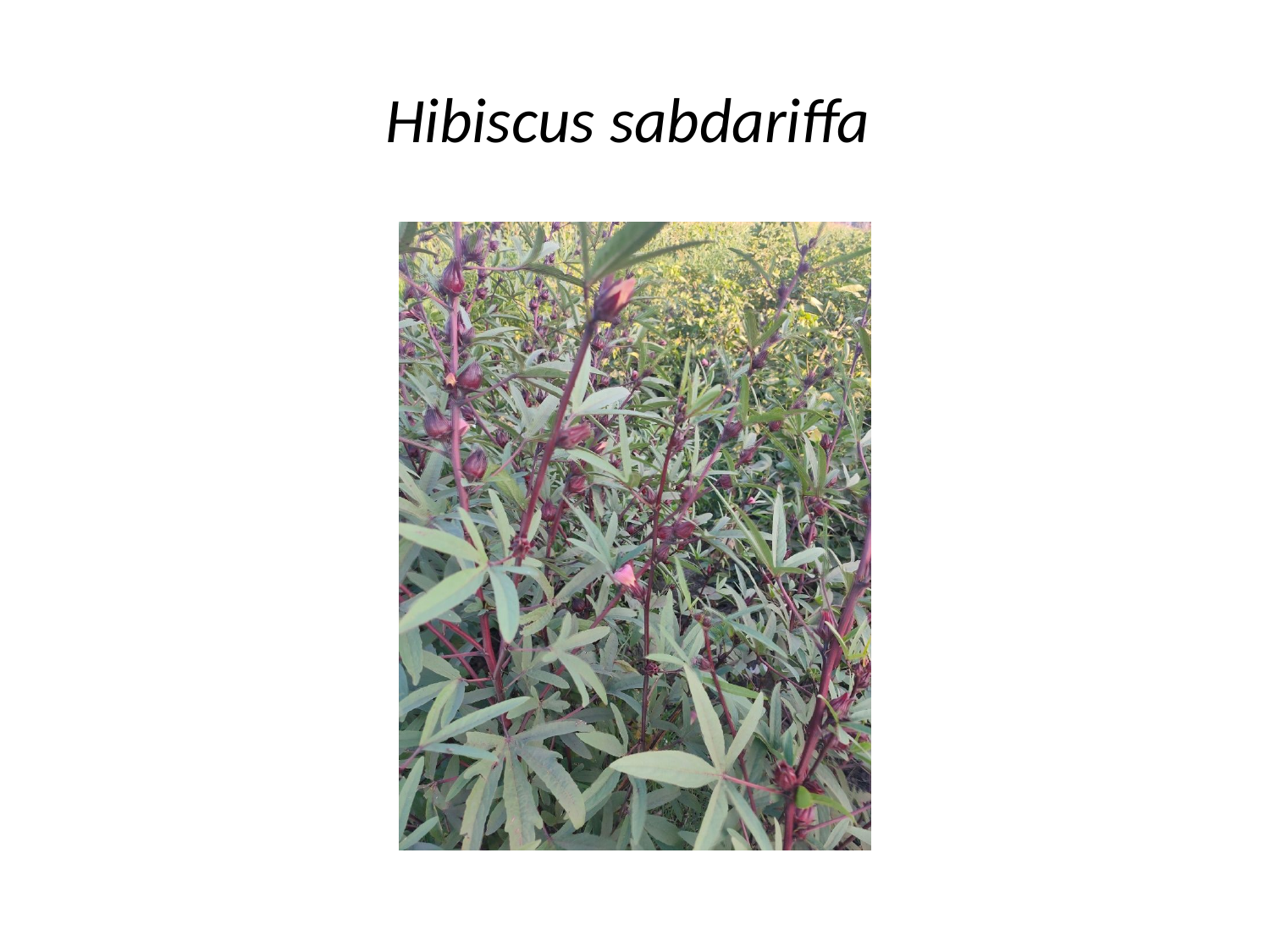

# Hibiscus sabdariffa

## Slide 16
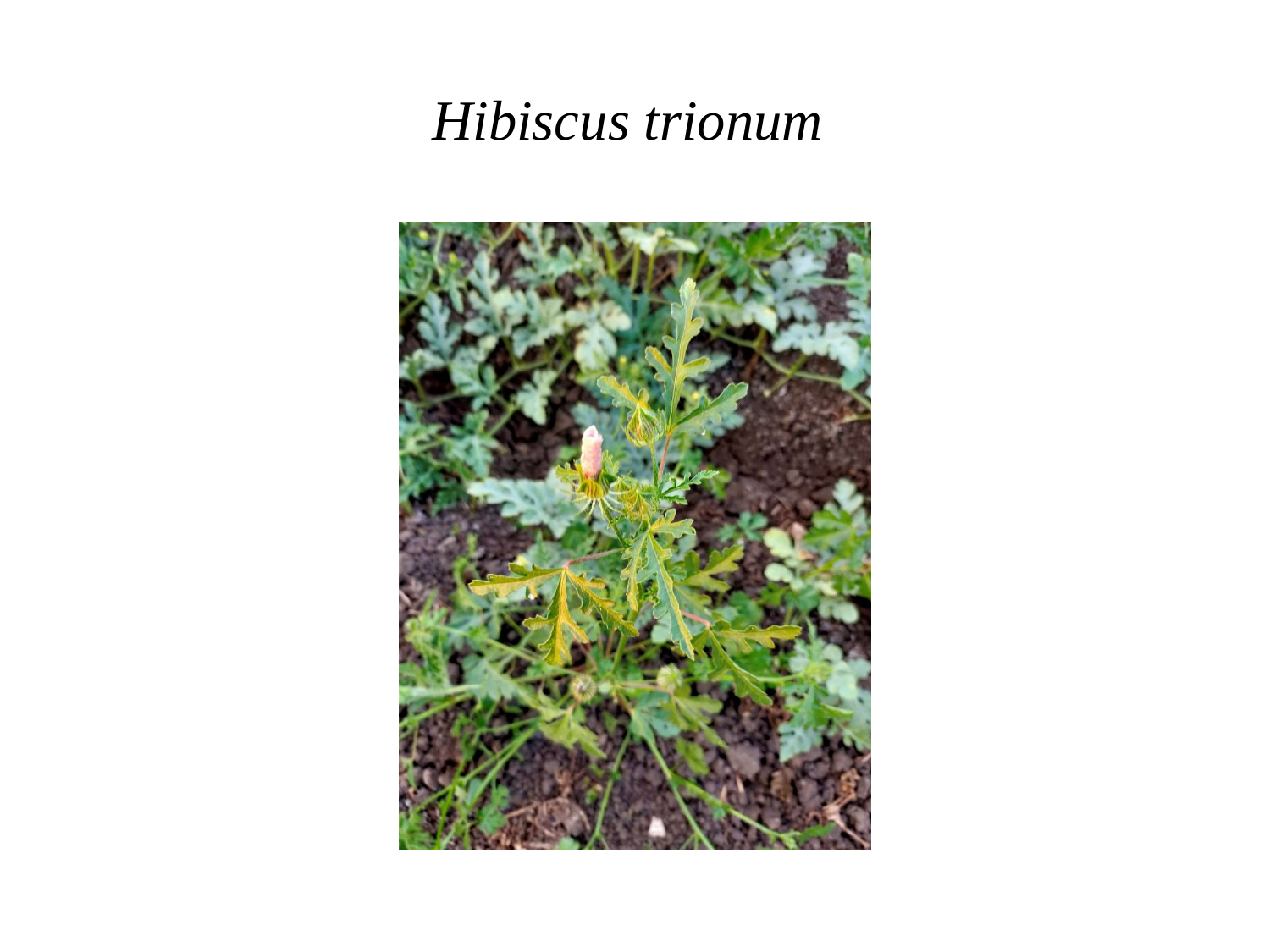

# Hibiscus trionum
